# Characteristics of Earthquake Cycles: A Cross‐Dimensional Comparison of 0D to 3D Numerical Models

**DOI:** 10.1029/2021JB023726

**Published:** 2022-08-19

**Authors:** Meng Li, Casper Pranger, Ylona van Dinther

**Affiliations:** ^1^ Department of Earth Sciences Utrecht University Utrecht The Netherlands; ^2^ Department of Earth and Environmental Sciences LMU Munich Munich Germany; ^3^ Department of Earth Sciences ETH Zurich Zurich Switzerland

**Keywords:** numerical modeling, earthquake sequence, cross‐dimensional comparison

## Abstract

High‐resolution computer simulations of earthquake sequences in three or even two dimensions pose great demands on time and energy, making lower‐cost simplifications a competitive alternative. We systematically study the advantages and limitations of simplifications that eliminate spatial dimensions in quasi‐dynamic earthquake sequence models, from 3D models with a 2D fault plane down to 0D or 1D models with a 0D fault point. We demonstrate that, when 2D or 3D models produce quasi‐periodic characteristic earthquakes, their behavior is qualitatively similar to lower‐dimension models. Certain coseismic characteristics like stress drop and fracture energy are largely controlled by frictional parameters and are thus largely comparable. However, other observations are quantitatively clearly affected by dimension reduction. We find corresponding increases in recurrence interval, coseismic slip, peak slip velocity, and rupture speed. These changes are to a large extent explained by the elimination of velocity‐strengthening patches that transmit tectonic loading onto the velocity‐weakening fault patch, thereby reducing the interseismic stress rate and enhancing the slip deficit. This explanation is supported by a concise theoretical framework, which explains some of these findings quantitatively and effectively estimates recurrence interval and slip. Through accounting for an equivalent stressing rate at the nucleation size h* into 2D and 3D models, 0D or 1D models can also effectively simulate these earthquake cycle parameters. Given the computational efficiency of lower‐dimensional models that run more than a million times faster, this paper aims to provide qualitative and quantitative guidance on economical model design and interpretation of modeling studies.

## Key Points

- Models with dimension reduction simulate qualitatively similar quasi‐periodic earthquake sequences with quantitative differences
- Reduced influence of velocity‐strengthening patches due to dimension reduction increases recurrence interval, slip and rupture speed
- We provide guidelines on how to interpret lower‐dimensional modeling results of interseismic loading and earthquake ruptures

## 1 Introduction

Destructive earthquakes every so often take us by surprise, because observations reveal a complex and opaque pattern of earthquake recurrence. Unraveling this pattern is challenging as the recurrence of large destructive earthquakes in nature is hardly observed. Some intermediate to large size earthquakes are observed to revisit the same fault (e.g., Chlieh et al., 2004; Prawirodirdjo et al., 2010; Segall & Harris, 1987). However, despite limited borehole data, these and most other natural observations are largely confined to the earth's surface, such that they remain indirect and at a distance to the hypocenter and thus often require inverse modeling to interpret. Earthquakes can also be generated quasi‐periodically in large‐scale laboratory experiments (e.g., McLaskey & Lockner, 2014; Rosenau et al., 2009) while these experiments are restricted to their millimeter to meter scale, such that they require a challenging upscaling step to interpret their findings. To complement our observations in nature and in laboratories, we need a quantitative description of the multi‐physics, multi‐scale processes governing fault slip. Numerical models are well‐suited to overcome these spatial‐temporal limitations and are thus important to improve our understanding of earthquake sequences and ultimately help to better estimate long‐term seismic hazard assessment.

Numerical models featuring different degrees of complexity in different dimensions have been used to simulate earthquake cycles. A fault can be modeled as simple as a single block slider connected to a spring. The spring provides the slider above the fault with a direct elastic response that mimics the response of the surrounding medium. We call this a zero‐dimensional (0D) model with a 0D fault point (e.g., Erickson et al., 2008; Gu & Wong, 1991; Madariaga, 1998; Ohtani et al., 2020). Similarly but more accurately, a fault can be modeled as a series of sliders connected to a series of springs in a row. This is a one‐dimensional (1D) model with a 1D fault line (Burridge & Knopoff, 1967; Petrillo et al., 2020). In both models the medium response is integrated directly onto the fault. However, it is also possible and common to mesh and model the surrounding medium explicitly, such that a rheologically more accurate and/or more heterogeneous response of the medium can be realized. A two‐dimensional (2D) model is required for a 1D fault line in this case (e.g., Barbot, 2019; Cattania, 2019; Herrendörfer et al., 2018; Lapusta et al., 2000; Van Dinther, Gerya, Dalguer, Mai, et al., 2013). To accommodate more on‐ and off‐fault complexity, including variations in fault properties or geometry along strike, a three‐dimensional (3D) model with a 2D fault plane is also prevalent (e.g., Barbot et al., 2012; Chemenda et al., 2016; Erickson & Dunham, 2014; Jiang & Lapusta, 2016; Lapusta & Liu, 2009; Okubo, 1989). In certain scenarios a so‐called 2.5D model is used for the sake of affordable computational cost, which approximately accounts for the effect of a finite fault width (e.g., Lapusta, 2001; Preuss et al., 2020; Weng & Ampuero, 2019). To do better justice to the large amount of earthquake cycle papers, we refer the reader to a white paper on future challenges for earthquake modeling (Lapusta et al., 2019) and an overview of benchmarked modeling codes provided in Erickson et al. (2020) and Jiang et al. (2022) for 2D anti‐plane and 3D settings, respectively. Generally, 3D models will produce results most representative for nature. However, given that they are still very time and energy consuming (Uphoff et al., 2017), simplified model setups are still largely adopted by many researchers and may be a very good choice to answer specific research questions (e.g., Allison & Dunham, 2018; Cattania, 2019; Romanet et al., 2020; Sathiakumar et al., 2020; Van Dinther, Preiswerk, & Gerya, 2019). A key reason for the need of such simplifications is the extremely high resolution required in both space and time, while at least exploring sensitivities in forward modeling studies (Lambert & Lapusta, 2021). On top of that, computational speed is particularly critical in situations where monotonous repetition of those forward models is required, for example, for inversion, data assimilation, physics‐based deep learning, uncertainty quantification, and when dealing with probabilities, such as for probabilistic seismic hazard assessment (e.g., Van Dinther, Künsch, & Fichtner, 2019; Weiss et al., 2019). However, also when trying to understand coupled multi‐physics or multi‐scale feedback these approximations can be really useful (e.g., Allison & Dunham, 2018; Lotto et al., 2019; Ohtani et al., 2019; Petrini et al., 2020; Van Dinther, Gerya, Dalguer, Corbi, et al., 2013). To optimize computing resources, researchers have to define suitable model complexities before and during their numerical simulations. Therefore it becomes a common concern to what extent lower dimensional models can reproduce nature when compared to 3D models. How are the observed differences in results attributed to the corresponding dimension reduction? And under what circumstances is this simplification justified?

These questions have not yet been systematically addressed. Nonetheless, several papers considered various aspects of this problem, especially via the comparison between 2D and 3D models. Lapusta and Rice (2003); Kaneko et al. (2010); Chen and Lapusta (2019) suggested ways to interpret their 2D results in more realistic 3D situations, such that they could be directly compared to 3D results. By doing this, they could compare velocity‐strengthening barrier efficiency in rupture propagation, seismic moment, and the scaling law for earthquake recurrence interval and seismic moment between 2D and 3D models in their studies. For the coseismic phase alone, the dynamic rupture community, conducting simulations with dynamic rupture models of one single earthquake, compared 2D and 3D results for benchmarking purposes. Harris et al. (2011) introduced two benchmark problems for dynamic rupture modelers where 3D simulations produced smaller ground motions (peak ground velocities) than in 2D simulations, in both elastic and elasto‐plastic scenarios. Similar 2D versus 3D comparisons focusing on coseismic rupture behavior as well as earthquake recurrence have also been made in the earthquake cycle community (e.g., Chen & Lapusta, 2009; Chen & Lapusta, 2019) where qualitative differences in earthquake magnitude and recurrence interval are discussed. However, these findings are not systematic and occasionally lack necessary theoretical support. Here we fill this gap by comparing earthquake cycle results across all dimensions from 0D to 3D, which includes comparisons for all phases of the earthquake cycle, that is, interseismic, nucleation, coseismic and postseismic phases.

We perform a systematic investigation of the limitations and advantages of models with different dimensions simulating cycles of characteristic earthquakes. By doing so, we compare physical characteristics and importance of different physical processes across dimensions both qualitatively and quantitatively. The aim of this paper is to serve as guidelines for modelers designing models and for all researchers interpreting results developed under necessary limitations. We first introduce the numerical method and the model setup of a strike‐slip fault under rate‐and‐state friction. The code package is validated and benchmarked by Southern California Earthquake Center (SCEC) Sequences of Earthquakes and Aseismic Slip (SEAS) benchmark problems BP1‐QD (Erickson et al., 2020) and BP4‐QD (Jiang et al., 2022) (see Supporting Information S1). Next, we systematically compare interseismic and coseismic characteristics of our models from 1D to 3D, summarizing and quantifying their advantages and shortcomings. The numerical results are explained and supported by a series of theoretical calculations. Finally the computational cost is compared. In the discussions, we first discuss under what conditions 2D models can substitute 3D models. Related issues on the model choices of this research, limitations and future improvements as well as possible applications are also discussed.

## 2 Methods

We exploit the flexibility of *Garnet*, a recently developed code library for the parallel solution of coupled non‐linear multi‐physics problems in earth sciences (Pranger, 2020). *Garnet* enables its users to formulate problems in a largely dimension‐independent way by defining a generic set of symbolic differential operators such as div and grad, which are then realized at compile‐time in the appropriate number of dimensions as concrete and performant compute kernels. Garnet implements the classical second‐order accurate staggered grid finite difference discretization of PDEs in space, and adaptive time stepping schemes of various orders of accuracy and other characteristics, all based on the linear multistep family of time discretizations. The library interfaces to PETSc (Balay et al., 2019a, 2019b, 1997) for linear and nonlinear solvers and preconditioners, to MPI (MPI Forum, 2015) for coarse scale distributed memory parallelism and intermediate scale shared memory parallelism, and to Kokkos (Edwards et al., 2014) (and in turn OpenMP, POSIX threads, or CUDA) for fine scale concurrency. In this section we further introduce the equations and algorithms that define our study.

### 2.1 Physics

Under the assumption of static stress transfer, the momentum balance equation reads

(1) ∇⋅σ=0,

where ***σ*** is the Cauchy stress tensor whose component *σ*_*ij*_ denotes the stress acting along the *x*_*j*_ axis on the plane that is normal to the *x*_*i*_ axis (*i*, *j* = 1, 2, 3). Both gravity and inertia are ignored in our models. Hooke's law relates stress rate $\dot{\sigma }$ to strain rate $\dot{\varepsilon }$ by

(2) $$\dot{\sigma }=2G\dot{\varepsilon }+\lambda \mathrm{Tr}(\dot{\varepsilon })I$$

with bulk modulus *K*, shear modulus *G*, Lame's constant *λ* ≔ *K* − 2*G*/3 and ***I*** identity tensor. $\mathrm{Tr}(\dot{\varepsilon })≔\;{\dot{\varepsilon }}_{kk}$ is the matrix trace. We assume infinitesimal strain rate $\dot{\varepsilon }$ as defined by

(3) $$\dot{\varepsilon }=\frac{1}{2}\left(\nabla v+v\nabla \right),$$

where ***v*** is the material velocity whose component *v*_*i*_ denotes the velocity in the direction *x*_*i*_ (*i* = 1, 2, 3). We use (*x*_1_, *x*_2_, *x*_3_) and (*x*, *y*, *z*) to refer to the three axes interchangeably.

For a fault with unit normal vector $\hat{n}$, the (scalar) normal stress *σ*_*n*_ (positive in compression) is given by the projection $\sigma_{n}=-\hat{n}\cdot \sigma \cdot \hat{n}$, the shear traction vector ***τ***_**s**_ by the projection $\tau_{s}=\sigma \cdot \hat{n}+\sigma_{n}\hat{n}$, the scalar shear traction *τ*_s_ by the Euclidean norm *τ*_s_ = ‖ ***τ***_**s**_‖, and finally the unit fault tangent $\hat{t}$ (which defines the orientation of the scalar fault slip *V*) by the normalization $\hat{t}=\tau_{s}/\tau_{s}$, such that $\tau_{s}=\hat{t}\cdot \sigma \cdot \hat{n}$. Further following Jiang et al. (2022), the fault is assumed to be governed by the rate‐and‐state friction law, which was initially proposed based on laboratory friction experiments by Dieterich (1979); Ruina (1983). We employ a regularization near zero slip velocity according to Rice and Ben‐Zion (1996) and Ben‐Zion and Rice (1997), so that the friction law that defines the relation between shear stress *τ*_s_ and normal stress *σ*_*n*_ on the fault is given by

(4) $$\tau_{s}=a\sigma_{n}arcsinh\left\{\frac{V}{2V_{0}}\exp\left[\frac{\mu_{0}}{a}+\frac{b}{a}\ln\left(\frac{\theta V_{0}}{L}\right)\right]\right\}+\eta V.$$

The “state” *θ* in turn is governed by the evolution equation

(5) $$\dot{\theta }=1-\frac{V\theta }{L},$$

corresponding to the so‐called “aging law” (Ruina, 1983). Symbols used in Equations 4 and 5 include the reference friction coefficient *μ*_0_, the reference slip rate *V*_0_, the characteristic slip distance *L*, and the parameters *a* and *b* that control the relative influence of direct and evolutionary effects, respectively. The fault is velocity‐weakening (VW) and potentially frictionally unstable when *a* − *b* < 0, and velocity‐strengthening (VS) and generally frictionally stable when *a* − *b* > 0. Finally, the parameter *η* used in Equation 4 refers to the “radiation damping term” used in the quasi‐dynamic (QD) approximation of inertia (e.g., Ben‐Zion & Rice, 1995; Cochard & Madariaga, 1994; Crupi & Bizzarri, 2013; Liu & Rice, 2007; Rice, 1993), which is employed in earthquake cycle simulations to reduce the computational costs. However, this is known to introduce qualitative and quantitative differences compared to fully dynamic (FD) modeling results (Thomas et al., 2014). The damping viscosity *η* = *G*/(2*c*_s_) is equal to half the shear impedance of the elastic material surrounding the fault.

### 2.2 Model Setup

Over the last decade, the SCEC has initiate various code comparison projects to verify numerical simulations on dynamic earthquake ruptures (e.g., Harris et al., 2009, 2018). The SEAS benchmark project (Erickson et al., 2020; Jiang et al., 2022), launched in 2018, is an extension to evaluate the accuracy of numerical models simulating earthquake cycles. We use this benchmark initiative to successfully verify the earthquake cycle implementation in *Garnet*, which is demonstrated in Supporting Information S1 and Jiang et al. (2022). To allow for a comparison with other existing and established implementations that are commonly used, we build our models based on the setup of SEAS benchmark problem BP4‐QD.

**Figure 1:**
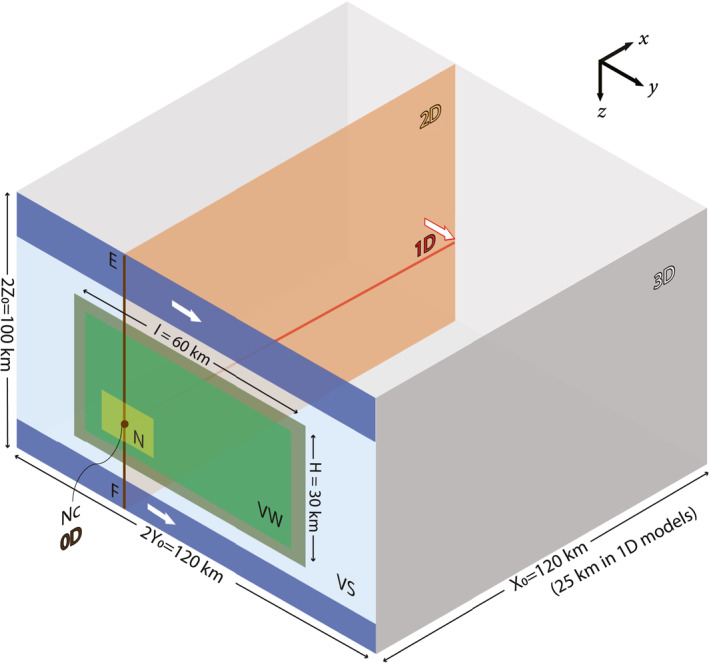
Numerical model setup of a vertical strike‐slip fault embedded in an elastic medium: 3D setup of Sequences of Earthquakes and Aseismic Slip benchmark BP4‐QD and its simplification to 2D, 1D and 0D. Only one side of the fault (half space *x* ≥ 0) is shown and modeled due to symmetry. “VW” and “VS” denotes the velocity‐weakening (VW) (light green) and velocity‐strengenthing (VS) (light blue) patches, respectively. The transition between VW and VS patches is shown in dark green. Tectonic loading regions at the top and bottom of the fault (dark blue) are subjected to constant velocities (white arrows). “N” denotes the predefined nucleation zone (yellow) with higher initial slip rate and shear stress, whose center is denoted as “Nc.” “EF” denotes a vertical line through “Nc.” Computational domain in 2D is reduced to *xz*‐plane (orange) with 1D fault line “EF” (brown). Computational domain in 1D is reduced to the *x*‐axis (red) with a 0D fault point “Nc” (brown). In this case tectonic loading is applied at the far‐away end with constant velocity (white arrow with red frame). Computational domain in 0D is fault point “Nc” without medium extent.

The BP4‐QD describes a planar vertical fault embedded in a homogeneous, isotropic linear elastic medium, observing the physics described in Section 2.1 (Figure 1). The *x*, *y*, *z* axes are directions perpendicular to the fault plane, along the strike and along the dip, respectively. Following Jiang et al. (2022), the fault condition is prescribed at *x* = 0. The central part of the fault is assumed to follow the rate‐and‐state friction formulation where a VW region is surrounded by a VS region. The top and bottom parts of the fault are not governed by rate‐and‐state friction and are instead subjected to a constant fault‐parallel loading velocity *V*_p_/2. The inherited frictional parameters *a*, *b*, *L* lead to a large nucleation size (∼12 km), such that it facilitated benchmarking under low resolution (500–1,000 m, Figure S3 in Supporting Information S1) with a reasonable computational load. We are aware that this setup allows for simple periodic earthquakes instead of smaller irregular ones but this simple earthquake sequence also facilitates the comparison over dimensions and make quantitative comparisons of some characteristic observations possible.

Due to the symmetry respective to the fault plane and the resulting anti‐symmetry of fault‐parallel motion, the motion at the fault is taken to be relative to a fictitious oppositely moving domain that is not modeled. The computational domain is thus limited to the half space *x* ≥ 0. Since this still proposes an infinitely large half space, the computational domain needs to be truncated to a finite domain when using a volumetric discretization. We use the computational domain **Ω**(*x*, *y*, *z*) = [0, *X*_0_] × [−*Y*_0_, *Y*_0_] × [−*Z*_0_, *Z*_0_] (Figure 1), where *X*_0_, *Y*_0_, *Z*_0_ are chosen sufficiently large to have negligible impact on the fault behavior (Jiang et al., 2022). The top and bottom boundaries *z* = ±*Z*_0_ are prescribed to move at the same constant loading velocity *V*_p_/2. The remaining three boundaries *x* = *X*_0_, *y* = −*Y*_0_, *y* = *Y*_0_ mimic the conditions at infinity and are set to be traction‐free. We show that the simulated earthquake sequences are converging in both interseismic and coseismic phases upon enlarging the medium thickness *X*_0_ and the difference is negligible when *X*_0_ > 40 km (Figure S1 in Supporting Information S1). The same parameter study is also implemented for *Y*_0_ and *Z*_0_ to achieve convergence (Table 1).

**Table 1:** Physical and Numerical Parameters

Parameter	Symbol	Value
Density	*ρ*	2.670 g/cm^3^
Shear wave speed	*c*_s_	3.464 km/s
Poisson ratio	*ν*	0.25
Shear modulus	*G*	32.0 GPa
Bulk modulus	*K*	53.4 GPa
Normal stress	*σ*_*n*_	50 MPa
Plate rate	*V*_p_	10^−9^ m/s
Width of rate‐and‐state fault	*W*_f_	80 km
Length of uniform VW region	*l*	60 km
Width of uniform VW region	*H*	30 km
Width of VW‐VS transition zone	*h*	3 km
Reference friction coefficient	*μ*_0_	0.6
Reference slip rate	*V*_0_	10^−6^ m/s
Characteristic slip distance	*L*	0.04 m
Rate‐and‐state direct effect	*a*	
VW		0.0065
VS		0.025
Rate‐and‐state evolution effect	*b*	0.013
Width of predefined nucleation zone “N”	*w*_*i*_	12 km
Distance of nucleation zone to boundary	*h*_*i*_	1.5 km
Initial slip rate		
Inside nucleation zone	*V*_*i*_	10^−3^ m/s
Outside nucleation zone	*V*_p_	10^−9^ m/s
Medium extent perpendicular to fault	*X*_0_	*40/80* /120^a^ km
Half fault extent along strike	*Y*_0_	60/*90* ^a^ km
Half fault extent along dip	*Z*_0_	50/*60* ^a^ km
Grid size	Δ*x*	500/*1000* ^a^ m

^a^ Numbers in italic are used in parameter studies.

The initial conditions are chosen to allow the fault to creep at the imposed slip velocity *V*_p_ in a steady state at *t* = 0 (Jiang et al., 2022), namely

(6) $$\theta \left(t=0\right)=\frac{L}{V_{p}},$$

and

(7) $$\tau_{s}\left(t=0\right)=a\sigma_{n}arcsinh\left\{\frac{V_{p}}{2V_{0}}\exp\left[\frac{\mu_{0}}{a}+\frac{b}{a}\ln\left(\frac{V_{0}}{V_{p}}\right)\right]\right\}+\eta V_{p}.$$

We additionally define a highly stressed zone “N” in the VW patch with higher initial slip velocity *V*_*i*_ (Figure 1) to ensure the first earthquake nucleates at that location when the computation starts. In this zone, the state variable *θ* keeps unchanged to achieve the high pre‐stress, namely

(8) $$\tau_{s}\left(\left(y,z\right)\in N,t=0\right)=a\sigma_{n}arcsinh\left\{\frac{V_{i}}{2V_{0}}\exp\left[\frac{\mu_{0}}{a}+\frac{b}{a}\ln\left(\frac{V_{0}}{V_{p}}\right)\right]\right\}+\eta V_{i}.$$

This helps us to better compare the coseismic behavior across dimensions. All physical and numerical parameters are summarized in Table 1.

### 2.3 Model Simplification by Progressive Elimination of Dimensions

In this work we take a structured approach to dimension reduction, eliminating first the lateral along‐strike dimension, then the vertical dimension, and finally the fault‐perpendicular dimension. Each of these steps are illustrated in Figure 1. For clarity, the assumptions and variables concerned in each dimension are summarized in Table 2.

**Table 2:** Simplifications in Different Dimensional Models

Model	Fault	Unknowns	Simplifications
3D	2D	*V*, *θ*; *v*_*x*_, *v*_*y*_, *v*_*z*_, *σ*_*xx*_, *σ*_*xy*_, *σ*_*xz*_, *σ*_*yy*_, *σ*_*yz*_, *σ*_*zz*_	No fault opening
2D	1D	*V*, *θ*; *v*_*y*_, *σ*_*xy*_, *σ*_*yz*_	+ Strike‐slip only, along‐strike invariant
1D	0D	*V*, *θ*; *v*_*y*_, *σ*_*xy*_	+ Along‐dip invariant
0D	0D	*V*, *θ*	+ Integral perpendicular to fault

In 2D, the model is simplified by excluding the along‐strike fault direction (denoted in orange in Figure 1). This means that the material and frictional properties, boundary and initial conditions are assumed to be homogeneous in this direction. That assumption thus omits the along‐strike heterogeneity introduced by the bounding VS patches as well. In this way, any half plane cutting the fault vertically may be taken as representative of the entire model. The computational domain can thus be reduced to **Ω**(*x*, *z*) = [0, *X*_0_] × [−*Z*_0_, *Z*_0_]. Furthermore, we omit the along‐dip motion *v*_*z*_ and only model the anti‐plane motion. As a consequence, only the *σ*_*xy*_ and *σ*_*yz*_ components of the stress tensor are required to be evaluated in this anti‐plane strain model. To allow a coseismic comparison we keep there the highly stressed nucleation zone defined in 3D and choose to model the plane cutting across this zone. The fault is collapsed to the line “EF” (denoted in red in Figure 1). Another common 2D perspective that models a horizontal plane cutting the fault includes the in‐plane strain assumption. While this configuration models a more complete set of momentum balance and elastic constitutive equations than the anti‐plane configuration we have chosen, the differences are only expected to manifest as a slightly modified elastic loading and corresponding changes in friction and nucleation size. We therefore choose to use the vertical 2D configuration that keeps the top/bottom loading regions for better comparison.

The simplified physical Equations (1), (2), (3) in 2D read:

(9) $$\begin{array}{cc} {\dot{\sigma }}_{xy} & =G\frac{\partial v_{y}}{\partial x},\\ {\dot{\sigma }}_{yz} & =G\frac{\partial v_{y}}{\partial z},\\ \frac{\partial \sigma_{xy}}{\partial x}+\frac{\partial \sigma_{yz}}{\partial z} & =0. \end{array}$$

In 1D, we further simplify the model by setting all variables invariant along dip in which case only the shear stress component *σ*_*xy*_ and the velocity component *v*_*y*_ remain. We thus lose the possibility to model spatial variations of frictional properties as the fault reduces to a 0D point at *x* = 0 in the computational domain **Ω**(*x*) = [0, *X*_0_]. We choose the fault “point” to be VW, corresponding to a location inside the predefined nucleation zone at “Nc” (denoted in red in Figure 1) to facilitate coseismic comparison. Furthermore, without an along‐dip fault extent, the original on‐fault tectonic loading from the top and bottom is no longer possible. Instead it is added at the far‐away boundary through a constant creeping rate there. To achieve a comparable interseismic stress rate inside the VW patch across dimensions, we adjust the domain size *X*_0_ so that the shortest distance between the VW patch and the creeping boundary is the same as in higher dimensional models. Namely, we set *X*_0_ equal to (*W*_f_ − *H*)/2.

The simplified physical equations in 1D read:

(10) $$\begin{array}{ccc} {\dot{\sigma }}_{xy} & = & G\frac{\partial v_{y}}{\partial x},\\ \frac{\partial \sigma_{xy}}{\partial x} & = & 0. \end{array}$$

In 0D, both the medium and the fault become the same point by eliminating the fault‐perpendicular dimension. In this model without medium extent, physical loading is impossible at any medium boundaries. Therefore a “driving force” that can be chosen arbitrarily (equivalent to loading at the fault point) has to be added to the system instead.

The simplified physical equation in 0D reads:

(11) $${\dot{\sigma }}_{xy}=-kV+{\dot{f}}_{d}$$

where *k* is the stiffness of the system and ${\dot{f}}_{d}$ is the applied driving force. This model will be further discussed in Section 4.2 where the equivalence of 1D and 0D models will be illustrated.

### 2.4 Numerical Algorithm

The nonlinear friction law Equation 4 and evolution law Equation 5 are solved in a point‐wise fashion using a Newton‐Raphson iteration for the slip rate *V* at a given stress ***σ***, given (6), (7), (8) (algorithm flowchart in Figure S2 in Supporting Information S1). The medium is closed with an essential velocity boundary condition $v=V\hat{t}/2$ on the fault (*x* = 0) and the remaining boundary conditions given in the two sections above.

We choose a spatial discretization that ensures that the smallest physical length scale in the rate‐and‐state friction model—the cohesive zone size Λ—is always well resolved. This cohesive zone size Λ (Day et al., 2005; Rubin & Ampuero, 2005) is given by

(12) $$\begin{array}{l} \;\Lambda =\Lambda_{0}\sqrt{1-\frac{V_{r}^{2}}{c_{s}^{2}}}\\ \Lambda_{0}=\frac{9\pi }{32}\frac{GL}{b\left(1-\nu \right)\sigma_{n}}, \end{array}$$

where *V*_*r*_ is the rupture speed and *c*_s_ is the shear wave speed. Λ_0_ is the upper limit of the cohesive zone size when *V*_*r*_ → 0. The dynamic cohesive zone size Λ shrinks with increasing rupture speed *V*_*r*_. We find that a high resolution is required for the seismogenic domain and its neighboring off‐fault area, while it is not required at medium to large distances to the fault. We improve computational efficiency by considering a grid that is statically refined (i.e., remaining fixed over time) near the VW zone. Refinement is realized by designing an orthonormal rectilinear (but not Cartesian) coordinate system that measures Euclidean space, and sampling this deformed coordinate system, rather than the Cartesian reference frame itself, at regular intervals. Differential operators are expressed in a general curvilinear coordinate system (see e.g., Simmonds, 1994) before discretization, a procedure that preserves the second‐order accuracy of the numerical method (Pranger, 2020).

We use adaptive time stepping to deal with the strong variation of the slip velocity and state variables in between interseismic and coseismic phases. The critically resolvable time scale is according to the evolution of the friction law (Equation 5). Following Lapusta et al. (2000), we let the time step Δ*t* be given by

(13) $$\Delta t=min\left\{\zeta \frac{L}{V_{max}},\left(1+\alpha \right)\Delta t_{old},\Delta t_{max}\right\}.$$

where *ζ* is a factor controlled by the material and frictional parameters (see calculation method in Lapusta et al., 2000). We also require the next time step not to be larger than (1 + *α*) times the former time step Δ*t*_old_ to avoid instability in the postseismic phase. A maximum time step size Δ*t*_max_ is further added to keep resolving the interseismic period in sufficient detail. We have used *α* = 0.2 and Δ*t*_max_ = 10^8^ s.

## 3 Results and Analysis

Following the simplifications summarized in Table 2 and Figure 1, this section compares and analyzes the 3D to 2D and 1D results, where the fault is modeled in 2D, 1D and 0D, respectively.

### 3.1 Interseismic Phase

**Figure 2:**
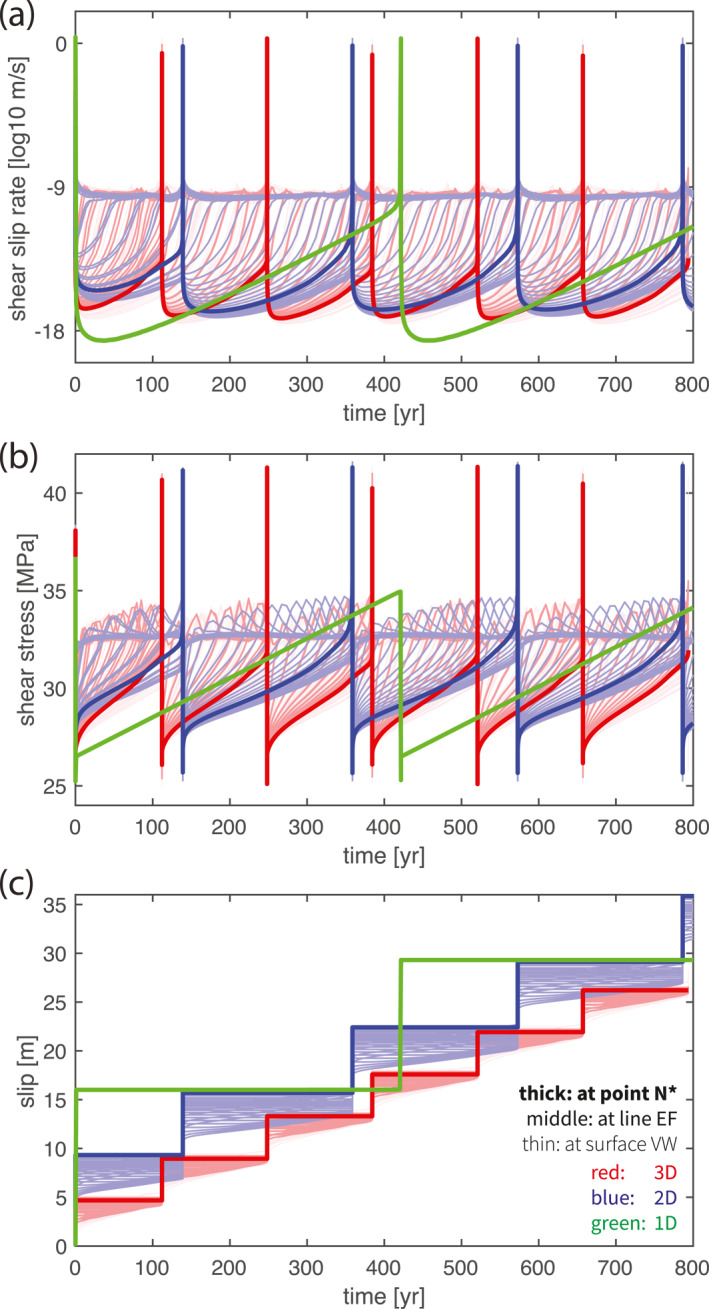
Comparison of the long‐term time series of (a) slip rate, (b) stress and (c) accumulated slip in 1‐3D models. The lines with different thicknesses and degrees of transparency are recorded at different locations on the fault, where the thick lines are recorded at the rim of the nucleation zone “N*” of the sixth earthquake, the semi‐thick lines along the line “EF” cutting across “N*” vertically and the thin lines elsewhere in the VW patch (see Figure 6).

Regardless of dimension, we observe quasi‐periodic earthquake sequences (Figure 2). In one earthquake cycle, shear stress is first accumulated from minimum 25 MPa to maximum 35–42 MPa during the interseismic phase and then released in an earthquake (Figure 2b). Accordingly, slip velocity also increases from locked rates of 10^−17^ m/s in 2D and 3D and 10^−20^ m/s in 1D to seismic rate 10^0^ m/s at the same time (Figure 2a). This similarity indicates the possibility of using lower dimensional models to substitute higher dimensional ones in earthquake cycle modeling.

**Figure 3:**
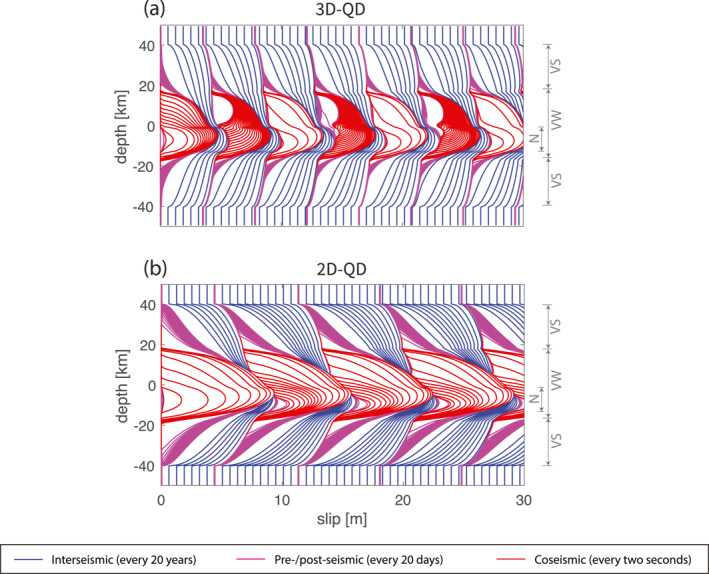
Cross‐dimensional comparison of cumulative seismic and aseismic slip. The cumulative slip profile of (a) the 3D model and (b) the 2D model, along the dip direction “EF” cutting across the predefined nucleation zone “N” (see Figure 1). “VW”, “VS”, “N” label the range of VW, VS and predefined nucleation zone. The interseismic phase is plotted every 20 years (blue), the pre‐ and post‐seismic phase every 20 days (magenta) and the coseismic rupture every 2 seconds (red). Note that the slip contour distortions around a depth of −1.5 and −13.5 km are introduced into these cumulative patterns by the predefined nucleation zone, whose properties increased the amount of slip in that zone for the first earthquake only.

By dimension reduction, simulated earthquakes become more characteristic (Figures 2 and 3). In 3D, all simulated earthquakes nucleate from one corner of the rectangular VW zone and rupture throughout it until the rupture front reaches the transition to the VS zone. However, not all earthquakes initiate from the same nucleation zone, as is suggested by the slip profile (Figure 3a). Rather, the nucleation location alternates between the top‐left and bottom‐right corners, resulting in a periodic cycle of two earthquakes with slightly different slip and recurrence interval. Similar results in 3D of two or more characteristic earthquakes repeating as a group have also been reported by Barbot (2019), where several possible mechanisms are suggested for this poorly understood phenomenon, including near‐stable condition, large geometrical aspect ratio and VW‐VS region interaction (see also Cattania, 2019; Chen & Lapusta, 2019). In 2D, earthquakes are more periodic because they all nucleate from the same down‐dip limit of the VW patch and rupture toward the up‐dip limit, instead of alternately nucleating from the top and bottom sides (Figure 3b). The earthquake size is also more identical with same recurrence interval. In 1D, we observe purely periodic, characteristic earthquakes of the same size (Figure 2). This trend is because with fewer dimensions, the interseismic loading pattern to the VW patch becomes simpler, so that the potential nucleation locations are also reduced. Earthquakes can potentially nucleate from four corners of the VW patch in 3D, but it reduces to two (top and bottom) in 2D and one in 1D. This demonstrate that as spatial dimensions are eliminated, the simulated results typically exhibit a simpler spatio‐temporal behavior.

From a quantitative point of view, simulated earthquakes reach larger slip and longer recurrence interval by dimension reduction (Figure 3). To quantify the difference in slip we compare the total slip (i.e., seismic slip + aseismic slip), because it is largely constant throughout the fault plane in one earthquake cycle. Total slip is also equal to the maximum coseismic slip, since the maximum is only achieved where the fault portion is fully locked in the interseismic period. This makes it, together with earthquake recurrence interval, good long‐term earthquake cycle characteristics. In 3D, we observe earthquakes with average total slip of ∼4.5 m and recurrence interval of ∼135 years (Figure 3a). In 2D, fault slips ∼6.8 m every ∼215 years, about 50% larger than in 3D (Figure 3b). In 1D, fault slips ∼13.3 m every ∼420 years, about three times as large as the 3D results and twice the 2D results (Figure 2c). Note that in calculation of these numbers we excluded the slightly larger first earthquake that initiated at the predefined nucleation zone.

We contribute the larger earthquakes simulated in lower dimensional models largely to a lower interseismic stress rate. During the interseismic phase, the VS patches are creeping at the plate rate so they do not accumulate stress. They only play a role in transferring the tectonic loading from the loading boundaries into the VW patch they surround. In other words, the VW patch is loaded directly by its surrounding VS patches rather than the loading boundaries, whether the bulk medium is simulated explicitly or not. This clarification is fundamental because in this way the VW patch in 3D is loaded from four sides, rather than only from the top/bottom where tectonic loading regions are located. While the VW patch in 2D is loaded from two sides, resulting in slower interseismic stress rate inside the VW patch and hence a longer period before the next earthquake can nucleate (thickest lines in Figure 2b). Given that the constant creeping rate in the VS patches is unchanged, the resulting larger slip deficit in the VW patch has to be made up by an earthquake with more slip. This is why larger earthquake slips are observed in lower dimensional models. Therefore these interseismic differences are largely explained by the reduced presence of VS patches due to dimension reduction. Quantitative calculations based on theoretical considerations, supporting the analysis above, will follow in Section 3.5.

That clarification also implies that the interseismic stress rate in the VW patch does not depend on the size of the VS patches *W*_f_ or the distance of the loading boundaries (*W*_f_ − *H*)/2, but on the size of the VW patch itself. The smaller the VW patch is, on average the faster the loading will be. This is because the average distance from a portion of the VW patch to the VW‐VS boundary is shorter. This explains why larger slip and longer recurrence interval are still observed in 1D even though the distance between the VW fault and the far‐away loading boundary *X*_0_ is already chosen to be (*W*_f_ − *H*)/2, the same as in higher dimensions (in Section 2.3). Using this concept we aimed to make the stress rate directly caused by the loading boundaries comparable to that in 2D and 3D models, but the actual stress rate proved to be inadequate. Therefore *X*_0_ has to be shortened to obtain higher stress rate, and finally to achieve similar earthquake slip and recurrence interval. Based on this idea and further theoretically considerations (Section 3.5) we propose a revised formulation of *X*_0_ in Section 4.2, where more similar results as in higher dimensional models are achieved.

### 3.2 Coseismic Rupture of the First Earthquake

**Figure 4:**
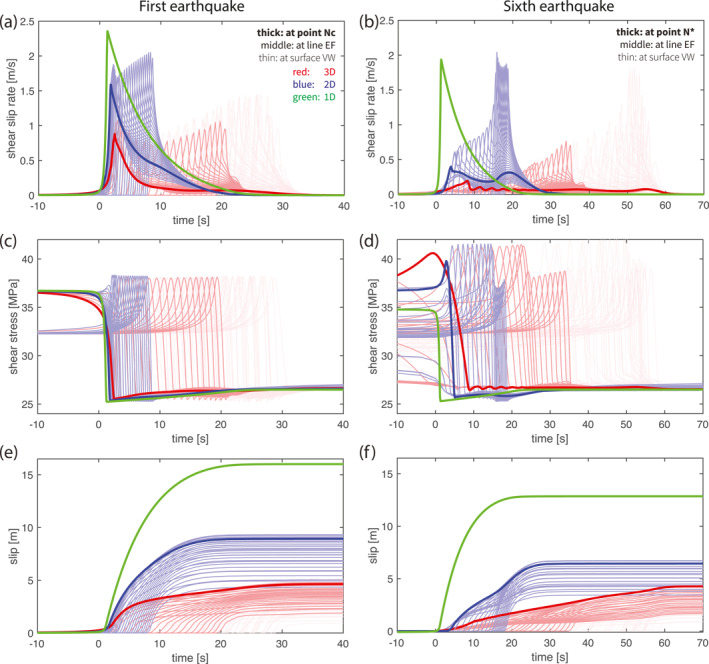
Comparison of the coseismic time series of (a, b) slip rate, (c, d) stress and (e, f) accumulated slip in 1‐3D models. The first earthquake is shown in (a, c, e), and the sixth earthquake is shown in (b, d, f), where origin time is set at the onset of the respective earthquake. The lines with different thicknesses and degrees of transparency are recorded at different locations on the fault, where the thick lines are recorded at the nucleation location “Nc” (the first earthquake) or “N*” (the sixth), the semi‐thick lines along the line “EF” cutting across it vertically and the thin lines elsewhere in the velocity‐weakening patch (see Figures 6a and 6c).

For the first earthquake (Figures 4a, 4c, and 4e), the source time function at all locations within the VW patch takes the shape of Kostrov's classic self‐similar crack solution (Kostrov & Das, 1988) with a short rise time and relatively long deceleration tail. As dimensions are reduced, the duration of the rise time decreases while the duration of the deceleration increases. The deceleration in 1D is the slowest, since the rupture does not interact with patches of different stress or strength properties that could decelerate it. For the same reason, it is impossible to observe rupture reflections in 1D. While the rupture reflection from the VW‐VS boundary in 3D is clearly observable as a second slip velocity peak (Figure 4a).

Despite this qualitative similarity, we compare slip velocity, rupture speed and stress drop for their quantitative differences across dimensions. Peak slip velocity and rupture speed are important earthquake characteristics that reflect the dynamic characteristics of a fracture. We observe that peak slip velocities reach the same order of magnitude of around 10^0^ m/s regardless of dimension, but they do increase by tens of percent in lower dimensional models (Figure 4a). In 3D, the peak slip velocity is initially ∼0.8 m/s in the predefined nucleation zone and gradually increases to its maximum of ∼1.5 m/s. In 2D, the peak slip velocity starts around ∼1.6 m/s and gradually increases up to ∼2.0 m/s. In 1D, the maximum slip velocity is ∼2.4 m/s. We connect this increase again to the reduced presence of VS patches due to dimension reduction. In 2D models, the 1D fault “line” represents a 2D fault plane in which the VW patch is extended infinitely long along strike in a 3D perspective (e.g., Andrews et al., 2007), whereas in 1D models the 0D fault “point” represents an infinitely large, fully‐VW 2D fault plane. In other words, the VS patches are removed from the dimensions that is not explicitly simulated, which would originally absorb energy from the rupture if the rupture would interact with them. More importantly, every portion of the fault along the not explicitly simulated direction ruptures at the same time as its simulated counterpart. Thus no fracture energy is consumed in those directions. The energy that is not consumed in these ways can instead be used to achieve higher slip velocities, as evident from the earthquake energy budget considerations in Kanamori and Rivera (2006).

**Figure 5:**
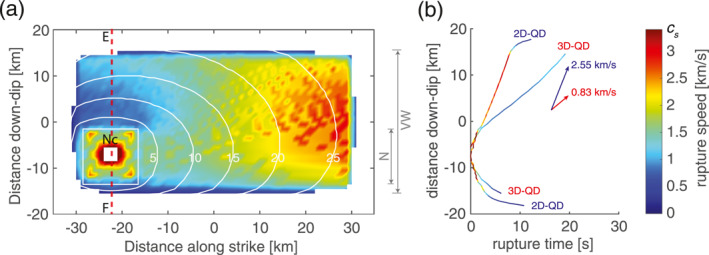
Comparison of coseismic rupture propagation. (a) The coseismic rupture speed of the first earthquake in 3D. The arrival time of the coseismic rupture front, which is measured when slip velocity reaching the seismic limit, is plotted every 5 seconds as contours. The central part of the fault plane is shown where white color means no seismic slip is observed. The red dashed line labels the observation line “EF” introduced in Figure 1. Note that no reliable rupture speed is measured at rupture onset (left white near “Nc”). (b) The coseismic rupture front arrival time along the vertical line “EF” in 2D and 3D. The line color indicates the rupture speed under the same color scale as (a). Lines end at where slip rates drop below seismic threshold. The average rupture speed in the middle of propagation (i.e., except during nucleation and arrest) is measured as stated.

Rupture speed across different dimensional models shows lager variation than peak slip velocity. In 3D, the total coseismic rupture lasts for ∼30 s. Rupture propagates faster in the horizontal direction than in the vertical direction and it experiences an acceleration in the last ∼10 s to reach near‐shear speed (Figure 5a). The rupture front takes ∼20 s to propagate along the vertical line “EF”, at a near‐constant speed of ∼0.83 km/s, except for the first several seconds and the arrest. In 2D, the rupture takes only ∼10 s to reach the up‐dip limit, starting from the same nucleation region (Figure 5b). Accordingly, the rupture speed of the stable part is ∼2.55 km/s, almost twice higher than in 3D. To explain these differences in rupture speed, the same considerations used to explain the differences in peak slip velocities are applied. In 2D models, no fracture energy needs to be overcome to rupture into the strike direction and hence more energy can be directed along dip, which allows the rupture to achieve higher speeds. This also shortens the rupture duration and leads to ruptures that propagate deeper into the surrounding VS patches compared to 3D models (Figure 5b). Given that the difference between 2D and 3D models occurs in the horizontal direction while the vertical direction remains identical, our results suggest that the (in)existence of the horizontal VS patches has influence on the coseismic rupture behavior inside the VW patch, even in the vertical direction. This is confirmed in additional models where a second rupture deceleration can be observed if the length of the VW patch is shortened to one fourth (see Section 4.1, Figure 9).

**Figure 6:**
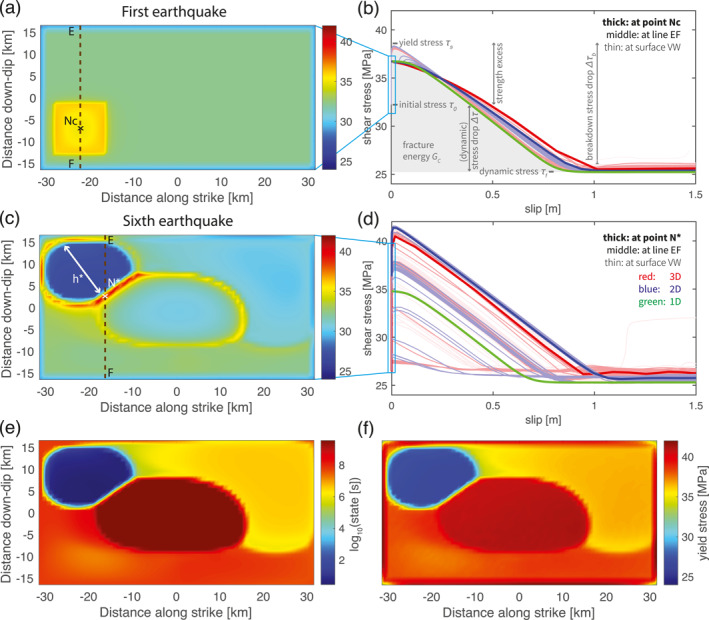
Cross‐dimensional comparison of (a, c) the initial stress and (b, d) the coseismic stress evolution w.r.t. slip in 1‐3D models for (a, b) the first earthquake and (c, d) the sixth earthquake. (a, c) The initial stress is measured when the maximum slip velocity reaches the seismic threshold. The nucleation size is denoted as h*. Due to the high prestress, the coseismic slip of the first earthquake begins from the center of the nucleation zone (denoted as “Nc”). Whereas the coseismic slip of the sixth earthquake begins at the rim of the nucleation zone (denoted as “N*”). (b, d) The lines with different thicknesses and degrees of transparency are recorded at different locations on the fault, where the thick lines are recorded at point “Nc” (the first earthquake) or “N*” (the sixth), the semi‐thick lines along the vertical line “EF” through it and the thin lines elsewhere in the velocity‐weakening patch (see panels a, c, respectively). (e) The initial state of the sixth earthquake. (f) The yield stress of the sixth event. The definitions of stresses and stress drops used in the text are labeled in panel (b).

Given the same initial condition, the stress drop and fracture energy of the first earthquake are comparable in all dimensional models, both inside and outside the pre‐stressed zone (Figure 6b). The stress drop Δ*τ*, that is, the stress difference between the start and the end of an earthquake, and the fracture energy *G*_c_, that is, the surface area below the stress w.r.t slip profile, are important earthquake parameters (see Figure 6b for more definitions of stresses and stress drops used below). Regardless of dimension and at all VW locations we first observe the shear stress increasing up to the yield stress and then it drops to a constant level corresponding to dynamic friction (Figure 4c). Both the yield stress and the dynamic stress are comparable across dimensions. Therefore the difference between the two (so‐called breakdown stress drop Δ*τ*_b_, i.e., strength excess + stress drop) is also similar. Notice that the initial stress increase is not as large when getting close to the nucleation zone and it is nearly zero inside it (thickest line in Figure 4c). This shows that the nucleation zone has to reach its yield stress before the coseismic phase, which is usually lower comparing to the maximum achievable yield stress elsewhere. After the stress drop, an immediate small stress increase is observed that is also similar in size across dimensions (Figure 4c). It is worth noting that the stress drop at different locations is achieved within a similar amount of slip (Figure 6b), regarded as the characteristic slip weakening distance *D*_c_ in a linear slip‐weakening friction formulation. After this distance, coseismic slip continues to accumulate until the earthquake arrests. The critical slip‐weakening distance varies from 0.8 to 1.1 m from 3D to 1D. Given the similar size of stress drop and slip‐weakening distance, the fracture energy *G*_c_ ≈ Δ*τ*_b_ *D*_c_/2 (Figure 6b) is also found to be comparable across dimensions and at all VW locations (with a minor increase from 1D to 3D).

The differences in stress drop and fracture energy across dimensions are minor. This is in line with expectations, since these earthquake parameters are considered to be largely controlled by the frictional properties and the normal stress (e.g., Rubin & Ampuero, 2005) that are homogeneous in this model. However, the modest systematic differences in, for example, the critical slip weakening distance that becomes shorter at lower dimensions, still indicates that the dynamics on the fault play a role in redistributing the earthquake energy budget, so that the stress drop and the slip weakening distance can change accordingly. This is more evident when the fault is shorted to one fourth its width where yield stress is observed decreasing while rupture propagates (see Section 4.1, Figure 9).

### 3.3 Nucleation Phase

A spontaneous nucleation phase is observed in later earthquakes that experience tectonic loading. To understand cross‐dimensional differences under more realistic initial conditions prevalent after the first earthquake, we also analyze the sixth earthquake. This earthquake is representative since earthquakes are essentially characteristic from the second onward.

Earthquake initiation somewhat differs across dimensions in how much aseismic slip is accumulated prior to nucleation and in the nucleation size h*. To understand this and to understand which fault plane locations are most comparable, we analyze interseismic slip velocity and shear stress evolution patterns (Figure 2). These patterns that depend on the distance between the observation point and the VS patches are qualitatively similar in all dimensional models. Faster loading occurs near the VS‐VW transition and these regions start to creep at plate rate the earliest. Slip becomes unstable when the creeping front propagates into the locked region up to the nucleation size h*. Nucleation then occurs in one of the four corners in the VW patch in 3D or one of the two ends in 2D. The nucleation size is observed to be roughly twice as large in 3D compared to the size in 2D (Figure 3). At the rim of this nucleation zone, highest shear stress is achieved due to the largest velocity gradient between creeping and locked zones. In the meantime, the inner nucleation zone yields and accelerates, which is accompanied by stresses dropping back to their steady‐state (Figure 6c). Based on whether the observation point is inside the nucleation zone, at the nucleation rim (e.g., point “N*” in Figure 6c) or outside the nucleation zone, similar loading and nucleating behavior is shared across dimensions, respectively (Figure 2). Inside the nucleation zone, faster slip velocity and stress accumulation rates are observed, both with a plateau at steady‐state before earthquake starts (middle to thin lines that are to the left and above the thickest line in Figures 2a and 2b). Outside the nucleation zone, at a point closer to the central VW patch that experiences slower loading, slip velocity and shear stress increase more slowly. This fault portion remains locked before the start of the next earthquake, that is, slip velocity is always below plate rate and shear stress below the aforementioned steady‐state stress level (middle to thin lines that are to the right and below the thickest line in Figures 2a and 2b). Only at the rim of the nucleation zone, can slip velocity and shear stress increase at a unique rate that allows for the earthquake to occur as soon as the plate rate and the fault strength are reached at the same time (e.g., thickest lines in Figures 2a and 2b). Since the seismic rate is achieved instantaneously, no aseismic slip is accumulated at this location during nucleation.

In 1D models with a 0D fault “point”, slip also immediately becomes seismic as soon as the shear stress reaches the interface strength and thus does not accumulate preceding aseismic slip. Therefore, such models mimic the rim of the nucleation zone in higher dimensional models (thickest lines in Figure 2). This is because, as we discussed above, the 0D fault “point” represents an infinite fully‐VW fault plane from a 3D perspective, on which earthquakes nucleate simultaneously at all locations as yield stress is reached at the same time. This location is where simulation results are best compared across dimensions and are further explored in theoretical calculations (Section 3.5).

### 3.4 Coseismic Phase of Later Earthquakes

An important consequence of interseismic loading is that it reshapes the initial stress (stress at the beginning of coseismic phase) and initial state to be heterogeneous (Figures 6c and 6e, also refer to panel b for the definition of below‐mentioned stress, stress drop and energy). Due to the variable distances to the VS patches and the nucleation process, different locations in the VW patch are loaded to a spatially variable level of initial stress and initial state. The nucleation zone has the lowest initial stress, whereas its rim has the highest values close to the yield stress (Figure 6c). The same holds for initial state except that a high state variable is also achieved in the center of the VW patch (Figure 6e). This is because during the preceding interseismic phase the central VW patch remains locked. According to Nakatani (2001)'s definition of interface strength $\left(\sigma_{n}\left[\mu_{0}+b\ln\left(\frac{\theta V_{0}}{L}\right)\right]\right)$, this region is healed to a much higher interface strength than its surrounding. Consequently, the subsequent coseismic phase exhibits characteristics that the first earthquake did not show.

Our dimensional comparison of the first earthquake regarding the rupture speed and slip velocity remains qualitatively valid (Figures 4b, 4d, and 4f vs. Figures 4a, 4c, and 4e) for the coseismic phase of later earthquakes. However, it is worth pointing out that the rupture speed is overall about 50% slower than the first earthquake, resulting in twice as long rupture duration in both 2D and 3D models (Figure 4b vs. Figure 4a). The peak slip velocity grows slowly at the beginning when the rupture is propagating into the central VW patch. The high interface strength suppresses its propagation into this patch and thus limits both rupture speed and peak slip velocity. Only once the rupture front has passed and is closer to the VW‐VS transition do the rupture speed and peak slip velocity increase sharply. Combining lower slip velocity and longer coseismic duration, the accumulated seismic slip is smaller in latter earthquakes than for the first earthquake (Figures 3 and 4f vs. Figure 4e). Smaller seismic slip is thus a result of the lower average initial stresses (and lower slip deficit) for spontaneously loaded earthquakes with respect to the highly stressed nucleation zone predefined for the first earthquake.

Given the same level of dynamic stress after the earthquake, the nonuniform initial stress field also results in a nonuniform stress drop Δ*τ* (Figure 6d). Additionally, the yield stress is spatially variable, making the breakdown stress drop Δ*τ*_b_ nonuniform as well (Figures 6d and 6f, also clearly visible in Figure 4d). The stress‐slip profile and fracture energy are thus no longer near‐identical throughout the VW patch as they are in the first event (Figure 6d vs. Figure 6b). Compared to the first earthquake, the yield stress becomes higher near the central VW patch and lower closer to the VW‐VS transition, making it lower when averaged over the whole seismogenic zone (Figure 6f). Fracture energy *G*_c_ varies accordingly: it increases near the center, decreases closer to the transition, and decreases on average. This illustrates the importance of tectonic loading for the coseismic rupture, as it modifies the initial stress, yield stress and energy profiles. Yield stress can thus no longer be simply defined by the frictional properties.

The 1D models, lacking the space for nucleation and dynamic rupture, never reach the initial and yield stress level higher dimensional models achieve in later earthquakes (Figure 4d). This makes them quantitatively dissimilar to 2D or 3D simulations in the coseismic phase, even from the aspect of mimicking the nucleation rim (thinkest lines in Figures 4b, 4d, and 4f vs. Figures 4a, 4c, and 4e).

### 3.5 Theoretical Considerations

To better analyze the similarities and understand the differences across dimensions, we utilize theoretical calculations that can estimate the aforementioned characteristic observables to the first order.

#### 3.5.1 Earthquake Cycle Parameters

We estimate earthquake recurrence interval and total slip (i.e., aseismic + seismic slip, maximum coseismic slip) by extending the 3D theoretical formulation in Chen and Lapusta (2019) to all other dimensions using the analytical crack models of Knopoff (1958) and Keilis‐borok (1959). Earthquake recurrence interval *T* can be estimated when it is known how much stress is accumulated and what the interseismic stress rate is, namely $T=\Delta \tau /\dot{\tau }$. Maximum coseismic slip *D*, which equals to the interseismic slip deficit, can be estimated from the aseismic slip accumulated on the surrounding creeping VS patches during the interseismic phase, namely *D* = *V*_p_ *T*.

To provide a reliable estimate of the interseismic stress rate and its maximum it is important to know which fault location is most representative for this purpose. This is important because the stress accumulation pattern is non‐linear and spatially variable (Figure 2), as explained in the description of the nucleation phase (Section 3.3). Give the nonuniform initial stress *τ*_*i*_ (Figure 6c) and the generally uniform dynamic stress *τ*_f_ as a starting level, the interseismic stress that needs to be accumulated Δ*τ* = *τ*_*i*_ − *τ*_f_ is thus not uniform. A similar spatial variation holds for the interseismic stress rate $\dot{\tau }$ (Figure 2b). Interestingly, the stress accumulates at an approximately linear rate at the rim of the nucleation zone, for example, at location “N*” in Figure 6c. Additionally, this location does not experience aseismic creep during the nucleation phase, as the slip becomes seismic immediately. These two observations make a straight‐forward theoretical calculation to estimate both recurrence interval *T* and maximum coseismic slip *D* feasible by analyzing the stress accumulation at location “N*.”

This location is at the distance of h* inside the VW patch since an earthquake can only nucleate when the creep penetrates this distance into the VW patch, where h* is the nucleation size. First, the interseismic stress accumulation is estimated by the stress drop Δ*τ*_dyn_, which is approximated from the stress difference between the two steady‐state friction level during the interseismic and coseismic phase (Cocco & Bizzarri, 2002)

(14) $$\begin{array}{cc} \Delta \tau_{\text{dyn}} & \approx \tau \left(V_{p}\right)-\tau \left(V_{\text{dyn}}\right)\\ & \approx \sigma \left[\mu_{0}+\left(a-b\right)\ln\left(V_{p}/V_{0}\right)\right]-\sigma \left[\mu_{0}+\left(a-b\right)\ln\left(V_{\text{dyn}}/V_{0}\right)\right]\\ & =\sigma \left(b-a\right)\ln\left(V_{\text{dyn}}/V_{p}\right), \end{array}$$

where dynamic slip velocity *V*_dyn_ is approximated as 1 m/s for simplicity. Second, the stress rate is calculated at the desired location that is at the distance of h* inside the VW patch (in 2D and 3D models, respectively, Rubin & Ampuero, 2005)

(15) $$\begin{array}{l} h_{2D}^{*}=\frac{2GLb}{\pi \sigma {\left(b-a\right)}^{2}}\\ h_{3D}^{*}=\frac{\pi^{2}}{4}h_{2D}^{*}=\frac{\pi GLb}{2\sigma {\left(b-a\right)}^{2}} \end{array}$$

for mode III deformation in our models. The factor *π* ^2^/4 comes from the stress intensity factor (SIF) that is different for different rupture front curvatures in 2D and 3D (Tada et al., 1973). The stress rate ${\dot{\tau }}_{h^{*}}$ at this location can be expressed as (Chen & Lapusta, 2019; Keilis‐borok, 1959; Knopoff, 1958)

(16) $${\dot{\tau }}_{h^{*}}=C\frac{GV_{p}}{\sqrt{r^{2}-{\left(r-h^{*}\right)}^{2}}}.$$

For a fault segment of half‐width *r* in 2D models or a circular fault of radius *r* in 3D models it has the same form with *C* a dimension‐dependent constant being either $C_{3D}=\frac{\pi \left(2-\nu \right)}{8\left(1-\nu \right)}=7\pi /24$ (Keilis‐borok, 1959) or *C*_2*D*_ = 1/2 (Knopoff, 1958). This expression is directly applicable to our 2D models with *r* = *H*/2. While in 3D models, taken into consideration that the width of VW patch *H* is shorter than its length *l*, we apply this expression to our rectangular fault by assuming *r* ≈ *H*/2. In 1D, the tectonic loading is applied from the far‐away boundary. In this case we replace the whole denominator $\sqrt{r^{2}-{\left(r-h^{*}\right)}^{2}}$ by *X*_0_, the distance between fault and the far‐away loading boundary, with *C*_1*D*_ = 1. Third, by combining the interseismic stress rate and coseismic stress drop together we approximate the recurrence interval *T* by

(17) $$T=\Delta \tau_{\text{dyn}}/{\dot{\tau }}_{h^{*}}=\frac{\left(b-a\right)\sigma }{CGV_{p}}\sqrt{r^{2}-{\left(r-h^{*}\right)}^{2}}\ln\frac{V_{\text{dyn}}}{V_{p}}.$$

Finally, the total slip *D*, or the maximum coseismic slip, is estimated by

(18) $$D=V_{p}T=\frac{\left(b-a\right)\sigma }{CG}\sqrt{r^{2}-{\left(r-h^{*}\right)}^{2}}\ln\frac{V_{\text{dyn}}}{V_{p}}.$$

**Figure 7:**
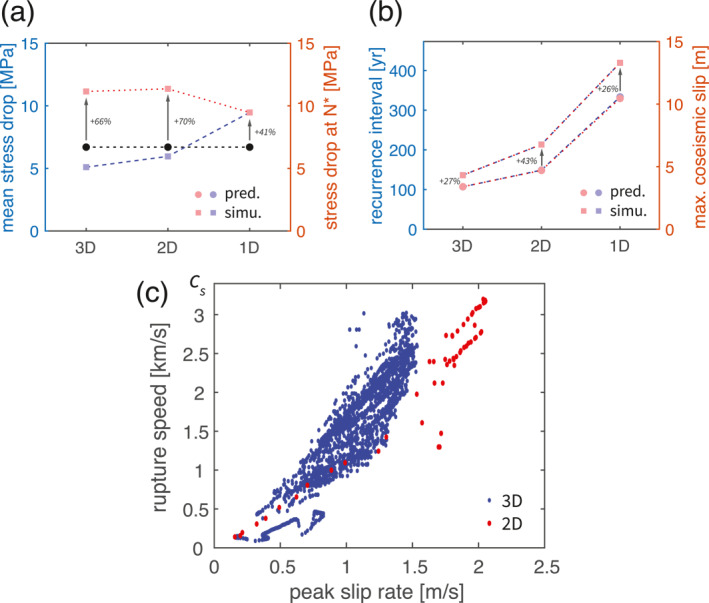
Comparison between theoretical predicted and numerically simulated results. (a) Comparison between theoretically predicted (circle) and numerically simulated (square) average stress drop (blue) and stress drop at location “N*” (red). The prediction is shared by both axis quantities and colored in black. The difference (in percentage) between calculated and simulated stress drop at location “N*” is labeled aside. (b) Comparison between theoretically predicted (circle) and numerically simulated (square) recurrence interval (blue) and maximum coseismic slip (red). Same labels as in (a). Note that the markers in blue and red are largely overlapped in this panel. (c) Interrelation between rupture speed and peak slip velocity in 3D (blue) and 2D (red) models. The local values are measured at different locations inside the VW patch.

The theoretically predicted recurrence interval and maximum coseismic slip values systematically underestimate the numerical simulations by about 30% but there is yet a qualitative agreement to simulations across all dimensions (Figure 7b). Especially, the theoretical prediction successfully captured the observed trend of increasing recurrence interval and coseismic slip a result of dimension reduction, with accurate relative changes from 3D to 2D and 1D. This agreement suggests that the essence of the cause is already captured in the simpler theoretical considerations and justifies our explanation that the larger coseismic slip is caused by the larger slip deficit during longer recurrence interval and the longer recurrence interval is caused by the lower interseismic stress rate. Further more, we notice that the relative difference is nearly identical between the recurrence interval and the total slip, indicating that the error in slip calculation (Equation 18 ) may be directly inherited from the recurrence interval calculation (Equation 17). The underestimation of the stress drop at location “N*” by stress drop Δ*τ*_dyn_ is a main contributor to this error (Figure 7a). Our simulations show that for the locations at the nucleation rim (point “N*” in Figure 6c) initial stress *τ*_*i*_ is notably higher than its surrounding. However, we notice that this underestimation of the accumulated stress is stronger than the underestimation of the final values (Figure 7a), indicating that the interseismic stress rate $\dot{\tau }$ is underestimated as well. This is due to the increased stress rate at the beginning and the end of the interseismic phase. At the beginning of the interseismic phase, it is increased by the effect of the postseismic slip. While near the end of the nucleation phase it is due to the expanding nucleation zone that creeps, introducing additional slip gradient (Figure 2b). Despite the errors, these theoretical considerations well explained the simulated earthquake cycle parameters and their trend with dimension reduction as a first order approximation.

#### 3.5.2 Coseismic Rupture Parameters

Unlike the recurrence interval and total slip, coseismic rupture parameters such as rupture speed and slip velocity vary across the fault. Our theoretical calculations cannot provide an absolute estimate of the rupture speed. However, both laboratory experiments (Ohnaka et al., 1987) and theoretical considerations (Ampuero & Rubin, 2008; Ida, 1973) suggest that the peak slip velocity *V*_peak_ and the rupture speed *V*_*r*_ are interrelated by

(19) $$V_{r}=\alpha_{r}V_{\text{peak}}\frac{G}{\Delta \tau_{b}},$$

where *α*_*r*_ is a factor on the order of 1. This positive correlation is confirmed by our simulations (Figure 7c). We measured on average *α*_*r*_ of 0.82 in 3D and 0.65 in 2D for the first earthquake respectively, which is similar to what Hawthorne and Rubin (2013) measured (0.50–0.65) in their 2.5D simulations. The lower value of *α*_*r*_ in 2D suggests that with dimension reduction higher slip velocity can be achieved under the same rupture speed.

Whereas the calculated stress difference from rate‐and‐state friction between the two steady states in the interseismic and coseismic phase (Equation 14) is independent of dimension and location, the stress drop Δ*τ* is not uniform across the simulated VW patch. Therefore that theoretical prediction only provides an estimation of the average stress drop (Chen & Lapusta, 2019)

(20) $$\bar{\Delta \tau }\approx \Delta \tau_{\text{dyn}}\approx \sigma \left(b-a\right)\ln(V_{\text{dyn}}/V_{p}).$$

The calculated average stress drop is slightly higher than the simulated results in 2D and 3D (Figure 7a). However, it is still satisfying as a first order approximation for both models given that the contribution of the changing state has been ignored. It is noticed that the 1D model has a higher simulated average stress drop. This is because the “average” loses its meaning in this case and the simulated value only represents where the earthquake nucleates in higher dimensional models (point “N*”). It is well expected that higher stress drop is achieved here following the explanation in Section 3.3 and the subsection above.

### 3.6 Computational Efficiency

**Figure 8:**
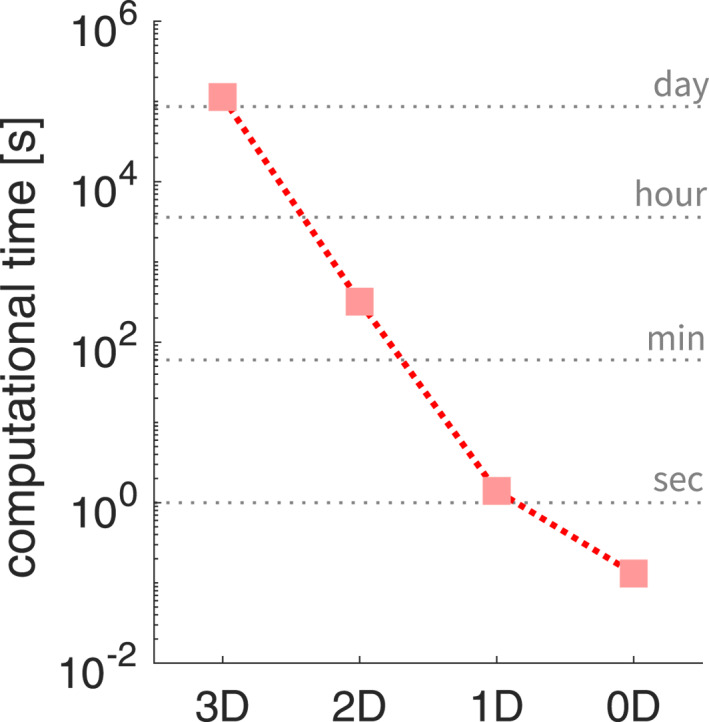
The average computational time of one earthquake cycle in 0D to 3D models, under the same resolution and domain size, with 12 CPUs Kokkos level parallelization.

Lower dimensional models are computationally more efficient without losing the qualitative characteristics and the ability to estimate certain earthquake parameters such as maximum slip velocity, maximum or average stress drop, and fracture energy. To evaluate the computational efficiency of each model we measure the average computational time per earthquake cycle (Figure 8). The 3D model takes 10^3^ times longer time than 2D and 10^5^ times longer than 1D. In the following discussions we will see that the 1D model can be further simplified to its 0D equivalent by removing the medium content (the *x* > 0 axis in 1D models). The 0D model will again save more than 90% running time compared to 1D, making it more than a million times faster than 3D models. Note that these computations do not use distributed memory and therefore ignore related parallel scaling issues.

## 4 Discussions

We are the first to systematically study and quantify similarities and differences in how models in different dimensions simulate earthquake sequences. While large‐scale parallel computing can be exploited to reduce the time to solution of 3D applications, this does not significantly lower the power consumption and consequently the monetary and environmental burden. Moreover, we find that the orders of magnitude difference of speed‐up by dimensional reduction are so large (Figure 8), and can be even larger when higher resolution is necessary, that they readily make the difference between being feasible for scientific and exploratory research or not. Hence lower dimensional models will likely remain essential for scientific exploration in the coming decades (Lapusta et al., 2019). Especially when the researcher's objectives fall into the scope of what the lower dimensional models can handle, they are encouraged to use them as they could be hundreds to millions times faster than a 3D model with the same resolution.

However, we should also acknowledge that there are research questions whose answers inherently require higher‐dimensional spatial or geometrical complexity. For example, rupture arrest in the missing dimension can never be captured in lower‐dimensional models, no matter if it is self‐arrested or due to the presence of VS patches. Temporally‐complex patterns of earthquake occurrence as well as partial ruptures reduce their existence at the same time. We are not aiming at finding substitutes for such cases but rather to present the essential differences that are apparent in the simplest setup. The differences between models of different dimensions presented in this paper will have no reason to disappear when more complicated setups are adopted. On the other hand, although 3D models are necessary for certain studies (e.g., Galvez et al., 2014; Madden et al., 2021; Ulrich et al., 2019; Wollherr et al., 2019), simpler models can always be a useful starting point of an exploration. These results should also serve as guidelines as to how to interpret the lower‐dimensional modeling results with their limitations ready in hand, rather than being regarded solely as restricting model simplifications to being adopted.

### 4.1 Under What Conditions Can 2D Models Substitute 3D Models?

**Figure 9:**
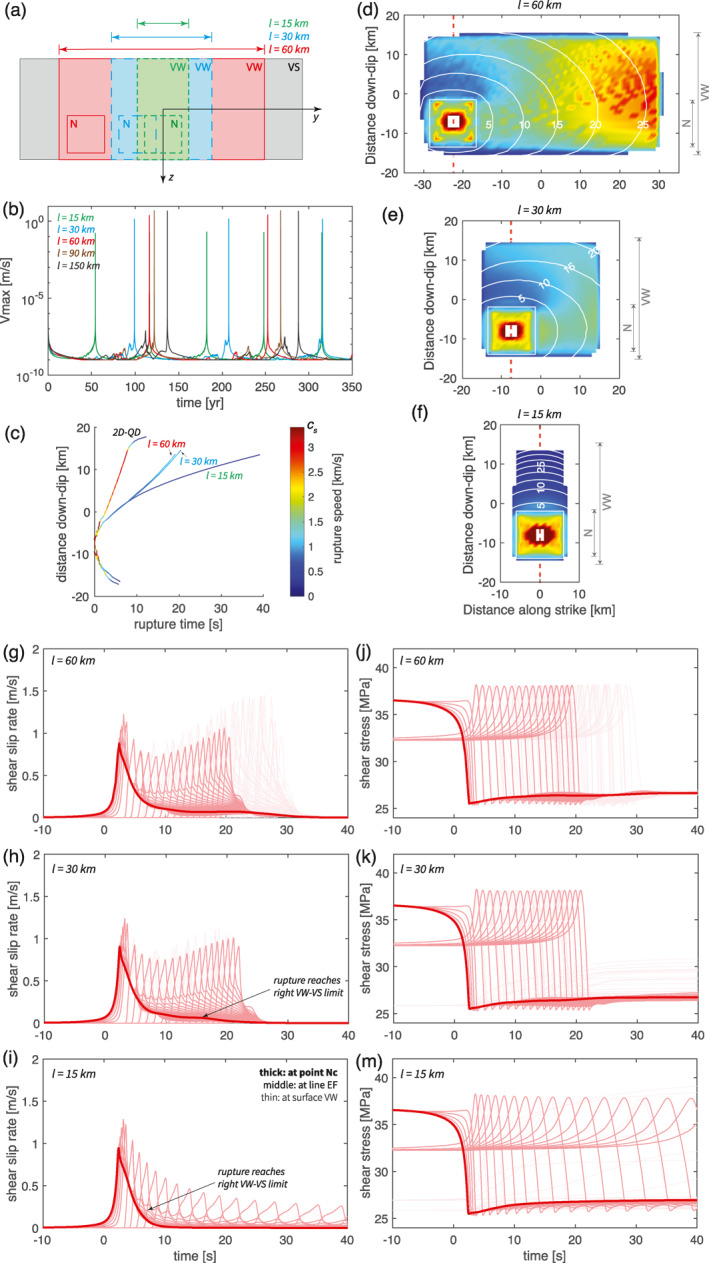
Comparison of the effects of fault length *l* (15–150 km) in 3D models: (d, g, j) 60 km, (e, h, k) 30 km, and (f, i, m) 15 km. (a) The varied velocity‐weakening patch sizes and varied locations of the predefined nucleation zone in three testing models with *l* from 15 to 60 km. (b) The maximum slip velocity in multiple earthquake cycles for models with *l* from 15 to 150 km. (d–f) The arrival time of the coseismic rupture front of the first earthquake, which is measured when slip velocity reaching the seismic limit. Only the central part of the fault plane is shown, where white color means no seismic slip is observed. Contours are plotted every 5 seconds. The red dashed line labels the observation line “EF” introduced in Figure 1. (c) The coseismic rupture front arrival time along the vertical line “EF” under the same color scale. Lines end at where no seismic slip is observed. The rupture time of the corresponding 2D model is plotted as reference. (g–i) The time series of slip velocity in the coseismic phase of the first seismic event, in which origin time is set at the onset of this event. The lines with different thicknesses and degrees of transparency are recorded at different locations on the fault, where the thick lines are recorded at point “Nc”, the semi‐thick lines along the line “EF” and the thin lines elsewhere (see Figure 1). (j–m) The time series of shear stress in the coseismic phase of the first seismic event, with the same line property.

We have summarized model similarities over dimensions as well as analyzed how model discrepancies due to dimension reduction explain the resulting differences. It is worth further exploring in which situations dimension reduction can be used without considerable side effects or when it should be avoided even if computational efficiency is a factor. To simplify the question, we restrict ourselves to the most common discussion point: under what conditions can a 3D model be substituted by a 2D model? Since along‐strike heterogeneities are ignored in the given dimension reduction assumption (Section 2.3), 3D models with different along‐strike features are simplified to the same 2D model. However, they originally simulate different earthquake sequences. We have chosen the VW patch length as one common along‐strike heterogeneity to analyze the role of this reduced dimension. We vary the VW patch length *l* and keep the VW patch width *H* fixed. By varying the VW patch length from 150 to 15 km, we change the aspect ratio from 5:1 to 0.5:1 (Figure 9). The fault (VW + VS patches) size and the computational domain (*X*_0_, *Y*_0_, *Z*_0_) are kept unchanged as well as the predefined nucleation zone as an initial condition, which is always set at the left bottom corner with fixed distance *h*_*i*_ to the VW‐VS boundary (Figure 9a). This configuration benefits the coseismic comparison along the vertical line “EF” crossing this zone (Figures 9c–9m) to our 2D simulations (Figures 4 and 5).

In the long term, longer VW patches result in longer recurrence intervals (Figure 9b). This is because the stress rate at the nucleation zone is lower comparing to a fault with a shorter VW patch. Given that the nucleation always starts from a corner of the rectangular VW patch, the nucleation zone in a longer VW patch is mainly loaded from three directions as the tectonic loading from the other horizontal direction is farther away. This is also supported by our theoretical considerations (see Section 3.5) where we assumed circular fault geometry in 3D and infinitely long fault in 2D. The elongated fault geometry deviates from the 3D assumption but is closer to the 2D one. Therefore longer recurrence intervals are to be expected. Consequently, by prolonging the VW patch length, we achieve longer recurrence intervals to fit better what is observed in 2D. In other words, higher aspect ratio faults in 3D are better represented by 2D models in the long term. However, even extending the 3D patch to 150 km still leads to shorter recurrence intervals comparing to what is observed in 2D (Figure 2), as interseismic loading remains more effective from three lateral sides than two.

On the other hand, a longer VW patch requires longer rupture propagation time along strike and thus longer coseismic duration, if the rupture speed remains unvaried (Figures 9d and 9e). As explain before, 2D models can be seen as 3D models where theoretically no time is required to rupture along strike. In this sense, a longer VW patch length is not preferred to fit the short coseismic duration observed in 2D. However, even the shortest coseismic duration, observed with aspect ratio 1:1, is still about 50% longer than 2D due to its low rupture speed. The rupture propagation time is not further shortened when the fault becomes even shorter. On the contrary, rupture speed is even largely decreased in the case with aspect ratio 0.5:1, resulting in a fairly long coseismic duration (Figures 9c and 9f). This speed change happens after the rupture front reaches the horizontal VW‐VS transition, confirming again that horizontal VW‐VS interaction can change vertical rupture speed. Accompanying the rupture speed reduction, the slip velocity and the stress drop are reduced at the same time (Figures 9g–9m). This is dissimilar to the observations in 2D (Figures 4a and 4c). From this aspect, a shorter VW patch length is not favored either. In other words, medium aspect ratio (close to 1:1) fault is better represented by 2D models in the coseismic phase. Additionally, if only what happens along the vertical line “EF” in 3D is taken into consideration when compared to 2D, then all models with aspect ratio higher than 1:1 can be accepted. This is because we notice that the rupture propagation along the vertical line “EF” does not change much with respect to the fault length when the aspect ratio is larger than 1:1 (Figure 9c). Nor do the slip velocity and coseismic slip change along this line (Figures 9d, 9e, 9g, 9h, 9j, and 9k).

To summarize, 2D models can better represent high aspect ratio faults in 3D for long‐term observations and medium‐to‐high aspect ratio faults for coseismic observations. Whereas for coseismic observations there are definitely inevitable qualitative differences in between. Our conclusion suggests that when using empirical scaling relations to interpret 2D results to a 3D perspective, it is crucial to assume a suitable aspect ratio according to the corresponding research objective. Wesnousky (2008) summarized 36 historical natural earthquakes and found that they have similar rupture width but varied rupture length, resulting in varied aspect ratio from 0.7 to 12. The analysis in this study, covering the range 0.5–5, can therefore be useful to refer to when comparing or validating 2D simulations to 3D natural observations.

### 4.2 Implication of Reducing Models to 1D or 0D

Our results and theoretical calculations suggest that 1D models reflect some key characteristics and thus can be used well to understand and quantify earthquake sequences under specific circumstances, which we discuss here. These implications from 1D models also hold for 0D models due to their mathematical equivalence. Since physical tectonic loading has to be removed in 0D models, an arbitrary “driving force” has to be added to the system instead (Section 2.3). To facilitate comparison, we can integrate the strain rate along the *x* direction in 1D models and use it to drive the 0D system. This is how the well‐known “spring‐slider” model is built (Burridge & Knopoff, 1967). Such a 0D model is mathematically equivalent to the 1D model. This is because the static momentum balance equation in 1D gives homogeneous shear stress in the medium. Combined with the boundary conditions, the time derivative of stress is given by

(21) $${\dot{\sigma }}_{xy}=G\frac{V_{p}-V}{X_{0}}.$$

Since this is an analytical derivation, the resulting model behavior is to remain the same. In this case we recommend to replace 1D models with 0D models, because they are more computationally efficient (Figure 8). Nevertheless, the explanation above no longer holds when the governing Equation 10 does not establish, including when heterogeneity, in‐elasticity and/or inertia are considered. In these more complex cases 1D models prevail in the ability of describing such physics (e.g., Pranger et al., 2022).

The domain size *X*_0_ in 1D and the arbitrary driving force ${\dot{f}}_{d}$ in 0D can be flexibly adapted to fit the earthquake cycle parameters. We have noted that setting the distance between the VW patch and the loading boundary *X*_0_ in 1D to be the same as in higher dimensions (*W*_f_ − *H*)/2 provides inadequate interseismic stress rate (Section 3.1). This is because tectonic loading is realized at the VW‐VS transition and it is neither dependent on *W*_f_ nor *H*. Relevant observations (Section 3.3) and theoretical considerations (Section 3.5) confirm that the 0D fault point mimics the nucleation rim in higher dimensional models that is located at a distance h* from the VW‐VS transition. By using the calculated stress rate (Equation 16) in 2D and 3D as the 0D “driving force” ${\dot{f}}_{d}$ in Equation 11, recurrence intervals of about 133 and 250 years are obtained. These are about 1.5% and 16% different from the real 3D and 2D simulations, respectively. This minor difference suggests that 0D and 1D models can be used to estimate both interseismic (e.g., earthquake recurrence interval) and coseismic (e.g., maximum coseismic slip) characteristics.

The commonly observed periodic slow slip events cannot be reproduced in 1D models with classical rate‐and‐state friction, as suggested by our explanation to the coseismic rupture characteristics (Section 3.2). In 1D the nucleation zone suddenly becomes infinitely large as soon as the 0D fault point starts to nucleate. This instability unavoidably leads to an earthquake (i.e., slip at seismic rate) instead of slow slip events. This inference is supported by a parameter study of hundreds of models in which no suitable frictional parameters could be found (Diab‐Montero et al., 2021). Slow‐slip events are only observed (slowly) decaying when the system stiffness is close to but smaller than the critical stiffness. Using the consideration that 0D fault point represents an infinitely large fully‐VW 2D fault, the infinite ratio of VW patch size (*H*) over nucleation size (h*) is known to lead to seismic slip rates (Herrendörfer et al., 2018; Liu & Rice, 2007). To produce slow‐slip events in 1D, additional damping needs to be present via, for example, rate‐dependent rate‐and‐state parameters (Im et al., 2020), two‐state variable rate‐and‐state friction behavior and/or additional spatio‐temporal complexities (Leeman et al., 2018). Not only slow‐slip events, any earthquake sequences including earthquakes that are not periodic, characteristic are hardly possible to be produced in 0D and 1D models, although they are to be expected most of the time in nature. The feature of the infinite VW fault dimension in 0D and 1D should be the first criterion to decide whether one should run a simulation in higher dimensions or not.

### 4.3 Implications for Other Model Setups

Our model was designed according to the SEAS benchmark BP4‐QD (Jiang et al., 2022) to maximize comparability, interpretability and reproducibility with a common setup featuring a simple recurrence pattern of a single earthquake rupturing the entire seismogenic zone instead of smaller ones with complex temporal patterns (Barbot, 2019; Cattania, 2019; Chen & Lapusta, 2019). Here we discuss several model setup adjustments, which largely shows that the conclusions drawn from our simulations can be generalized to a broader context.

We have investigated the similarities and differences in models of different dimensions using a FD approach to extend the applicability of our statements. Our conclusions still largely hold with minor quantitative variations. However, we also found qualitative differences in coseismic characteristics that demand a deeper discussion via the comparison between QD versus FD models, which we for clarity referred to a follow‐up paper (Li et al., 2021).

Tectonic loading is typically applied in two different ways: directly on the fault plane (e.g., Kaneko et al., 2011) or indirectly at the far‐away boundaries (e.g., Herrendörfer et al., 2018). Both types have been adopted by studies for different research purpose. We adopted tectonic loading at the top/bottom of the fault plane for 2D and 3D models following BP4‐QD, but at the far‐away boundary for 1D models due to dimensional restriction. To test the influence in the interseismic phase we applied tectonic loading conditions (a) only on fault surface at top/bottom region with fixed fault width, (b) only on far‐away boundary surface, (c) both (a) and (b). We modeled in 2D with gradually enlarged computational domain (Table S1 in Supporting Information S1). We find that the recurrence interval converges to a set value as the computational domain is enlarged and is hardly affected by the type of loading when the computational domain is large enough. This invariance with respect to loading condition is supported by our theoretical calculations (Section 3.5). Because there we explained that the main loading force to the locked VW patch is from its surrounding creeping VS patches. No matter which type of loading is applied, the stress rate inside the VW patch is largely defined by its own dimension and independent of the size of the VS patches or the fault as a whole (Equation 16). Naturally the velocity gradient perpendicular to the fault contributes to the loading process as well, but it is minimized for large enough computational domain where on‐fault loading becomes dominant. During the coseismic period, the way in which tectonic loading is applied does not influence results because of the short duration. Therefore both the interseismic and coseismic characteristics are not sensitive to what kind of loading boundary condition is applied. Comparison in the SEAS benchmark BP4‐QD of different modeling groups demonstrated the same idea: numerical results generally agreed with each other when computational domain was large enough, where for the numerical method's convenience, either stress‐free or constant‐moving boundary condition is chosen at far‐away boundaries (Jiang et al., 2022).

As for the initial condition, we have adopted a predefined highly‐stressed zone within the VW patch following BP4‐QD. Since the later earthquakes do not necessarily occur from the same location, this predefined zone facilitated the quantitative coseismic comparison across dimensions by forcing the first earthquake to nucleate from this same region. It is suggested by some former studies that initial conditions have little effect on subsequent earthquakes (e.g., Allison & Dunham, 2018; Takeuchi & Fialko, 2012), therefore this special initial condition should not harm our findings in terms of earthquake cycle characteristics as well as nucleation behavior. In this study we did notice that the accumulative slip contour distortions around a depth of −1.5 and −13.5 km are introduced by the predefined nucleation zone, whose properties increased the amount of slip in that zone for the first earthquake (Figure 3). However, for non‐accumulative variables no influence from the initial condition is observed in later earthquakes. Nevertheless, the first earthquake is not completely characteristic in an earthquake cycle even though some qualitative characteristics are still shared by later earthquakes. This also becomes apparent in the comparison to the sixth earthquake.

## 5 Conclusions

In this paper, we addressed a common concern of numerical modelers: how complex should my model be to answer my research question? Will dimension reduction qualitatively and quantitatively affect my results? And how? For this purpose we have systematically investigated different dimensional models from 0D to 3D in terms of their interseismic and coseismic characteristics and computational time for earthquake sequences and individual QD ruptures.

Our results demonstrate that, when 2D or 3D models produce quasi‐periodic characteristic earthquakes, their behavior is qualitatively similar to lower‐dimension models The stress accumulation pattern is much the same when observed at the rim of the nucleation zone. As for the earthquake cycle parameters, lower dimensional models produce longer recurrence intervals and hence larger coseismic slip. We observe that the VS patches play a crucial role in causing differences in the interseismic phase, because tectonic loading is effectively realized at the VW‐VS transition by the velocity contrast between the creeping VS patches and the locked VW patch. As VS patches are removed when fault dimension is reduced, their absence reduces the interseismic stress rate inside the VW patch and thus increases the recurrence interval. The larger slip deficit built in this period leads to a larger coseismic slip.

In the coseismic phase, we find that certain earthquake parameters such as the stress drop and fracture energy can be accurately reproduced in each of these simpler models, because they are mainly governed by material frictional parameters. This finding is especially valid for the first earthquake without physical tectonic loading. For later earthquakes, the statement is only true on average of the VW patch. This is because the initial stress, yield stress and effective slip weakening distance can change due to tectonic loading and earthquake history. For the coseismic rupture parameters, lower dimensional models generally produce higher maximum slip velocities and higher rupture speeds in lower dimensional models. Furthermore, we demonstrate that the interaction at the VW‐VS transition can modify rupture speed, which is another crucial role the VS patches play in the coseismic phase. We find that the vertical rupture speed along the vertical direction in 3D is slower compared to 2D. It can be further slowed down when the fault length is shortened even more, suggesting that the vertical rupture behavior is influenced by horizontal frictional properties.

The aforementioned findings are supported by our theoretical calculations, which confirm that geometric differences due to dimension reduction influence the interseismic loading and finally affect the subsequent coseismic phase. Through accounting for an equivalent stressing rate at the nucleation size h* into 2D and 3D models, 0D and 1D models can also effectively estimate earthquake cycle parameters such as recurrence interval and total slip. These theoretical considerations can be generally applied to other earthquake cycle models as well.

Based on these differences and similarities, we have analyzed under what condition 3D models can be substituted by 2D models by focusing on the aspect ratio of the VW patch. Our results present that 3D models with longer fault length have longer recurrence interval, which fits better 2D observations in the long term. On the other hand, shorter fault length requires shorter rupture propagation time along strike, which fits better 2D observations in the coseismic phase.

Finally, we highlight the power of lower dimensional models in terms of their computational efficiency. We find that under the same (relatively low) resolution 3D models require 10^3^ times longer computational time than 2D, 10^5^ times longer than 1D and 10^6^ times longer than 0D models. Therefore dimension reduction can not only relieve the heavy energy‐consuming simulations, but also improve the efficiency of projects that require monotonous repetitions of forward models. This paper may serve as guidelines to check in simplified models what results can be expected to be accurately modeled as well as what physical aspects are missing and how they are related to the discrepancies observed in the results.

## Supporting information

Supporting Information S1

## Data Availability

The data produced and analyzed in this study are available via https://doi.org/10.5281/zenodo.6009287.
