# Supplementary material for: Characteristics of Earthquake Cycles: A Cross‐Dimensional Comparison of 0D to 3D Numerical Models
Source: J Geophys Res Solid Earth. 2022 Aug 19;127(8):e2021JB023726. doi: 10.1029/2021JB023726 (PMC9539514; doi:10.1029/2021JB023726)
Supplement: Supplementary file 1 — Supporting Information S1 [file JGRB-127-e2021JB023726-s001.pdf]

Supporting Information for "Characteristics of earthquake cycles: a cross-dimensional comparison of 0D to 3D numerical models"

Meng Li[1], Casper Pranger[2,3], and Ylona van Dinther[1]

[1]Department of Earth Sciences, Utrecht University, Utrecht, Netherlands

[2]Department of Earth and Environmental Sciences, LMU Munich, Germany

[3]Department of Earth Sciences, ETH Zurich, Switzerland

Contents of this file

1. Text S1
2. Figures S1 to S3
3. Table S1

Text S1. Code validation

We have validated the code library *Garnet* and our models through the published results of SCEC benchmarks BP1-QD/-FD (Erickson et al., 2020) and the recently submitted results of BP4-QD (Jiang et al., 2022), which also includes our own results.

Both the long term and coseismic behaviors match well with other modelers participated in the 3D QD benchmark BP4-QD (Fig. S3). The long-term shear stress and slip rate time series from *Garnet* (finite difference method) agree very well with the results

---

Corresponding author: Meng Li, (m.li1@uu.nl)

from other methods including boundary element method (S. Barbot, Unicycle, Barbot, 2019), finite element method (D. Liu, EQsimu, Liu et al., 2020), and spectral boundary integral method (J. Jiang and V. Lambert, BICyclE, Lapusta et al., 2000; Lapusta & Liu, 2009). Our results lie well in the center of all the models, indicating the validity of *Garnet* for usage in earthquake cycle modeling. The comparison between *Garnet*, EQsimu and BICyclE of the coseismic rupture propagation also reveals the consistency of the three numerical methods in quasi-dynamic earthquake rupture modeling. We notice from the rupture contour curvature that *Garnet* has a horizontal rupture speed larger than BICyclE, but smaller than EQsimu. This discrepancy might come from the boundary condition that is slightly differently applied in each numerical method.

In the 2D QD benchmark BP1-QD (Fig. S4), our results show a high similarity in terms of recurrence period, total slip and cumulative slip profile, compared to the results of other models participating in the same benchmark (cf. fig. 3 in Erickson et al., 2020). This indicates the reliability of *Garnet* in solving the benchmark. A further comparison of slip rate and shear stress between *Garnet* and BICyclE reveals that the evolution pattern of slip rate and shear stress of both models overlap well in the long term, except for a delay observed in BICyclE comparing to *Garnet*. It is worth to mention that the earthquake sequence simulated by *Garnet* is slightly smaller in terms of total slip, recurrence time and maximum slip rate compared to BICyclE. The surface reflection also comes later. This is due to that these two models have been implemented with different boundary conditions. Although both were performed in the same domain size of 160 km depth, the BICyclE model has a periodic boundary condition. Since the interaction between the neighboring seismogenic patches may influence the tectonic loading during interseimic period, our

result with a constant loading bottom boundary is more reliable in this aspect, which is also verified by the comparison with other models in Erickson et al. (2020) (cf. fig. 5, 6 therein).

All external data used in this section and Fig. S3-4 are available via SCEC benchmark platform `https://strike.scec.org/cvws/seas/` (Erickson et al., 2020; Jiang et al., 2022).

References

Barbot, S. (2019). Slow-slip, slow earthquakes, period-two cycles, full and partial ruptures, and deterministic chaos in a single asperity fault. *Tectonophysics*, *768*, 228171.

Erickson, B. A., Jiang, J., Barall, M., Lapusta, N., Dunham, E. M., Harris, R., ... Wei, M. (2020). The community code verification exercise for simulating sequences of earthquakes and aseismic slip (seas). *Seismological Research Letters*, *91*(2A), 874–890.

Jiang, J., Erickson, B. A., Lambert, V. R., Ampuero, J.-P., Ando, R., Barbot, S. D., ... van Dinther, Y. (2022). Community-driven code comparisons for three-dimensional dynamic modeling of sequences of earthquakes and aseismic slip. *Journal of Geophysical Research: Solid Earth*, *127*(3), e2021JB023519. doi: https://doi.org/10.1029/2021JB023519

Lapusta, N., & Liu, Y. (2009). Three-dimensional boundary integral modeling of spontaneous earthquake sequences and aseismic slip. *Journal of Geophysical Research: Solid Earth*, *114*(B9).

Lapusta, N., Rice, J. R., Ben-Zion, Y., & Zheng, G. (2000). Elastodynamic analysis for slow tectonic loading with spontaneous rupture episodes on faults with rate-and

state-dependent friction. *Journal of Geophysical Research: Solid Earth*, *105*(B10), 23765–23789.

Liu, D., Duan, B., & Luo, B. (2020). Eqsimu: a 3-d finite element dynamic earthquake simulator for multicycle dynamics of geometrically complex faults governed by rate- and state-dependent friction. *Geophysical Journal International*, *220*(1), 598–609.

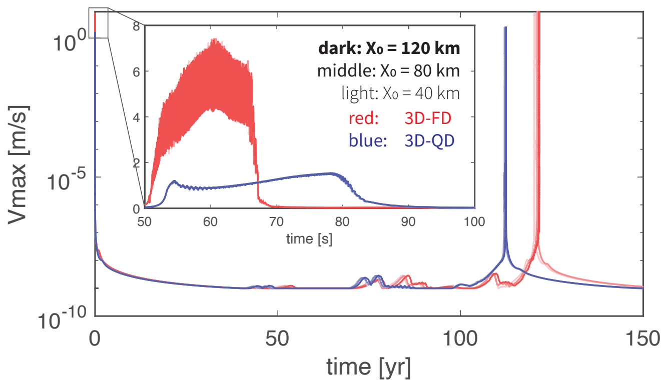

Figure S1. Influence of computational domain size: comparison of long-term and coseismic maximum slip velocity with various medium thickness X_0 choices in 3D models. The inner panel shows the coseismic zoom-in to the first earthquake event.

Table S1. Influence of tectonic loading realization: Recurrence interval (yr) under different tectonic loading conditions and computational domain size in 2D QD model.

Medium extent X_0	Loading condition (a)	(b)	(c)
80 km	104.0	125.5	104.0
40 km	104.0	128.0	104.0
20 km	101.5	118.5	101.0
10 km	103.0	87.5	86.0

(a) only on fault surface at top/bottom region with fixed fault width,
(b) only on far-away boundary surface,
(c) both (a) and (b).

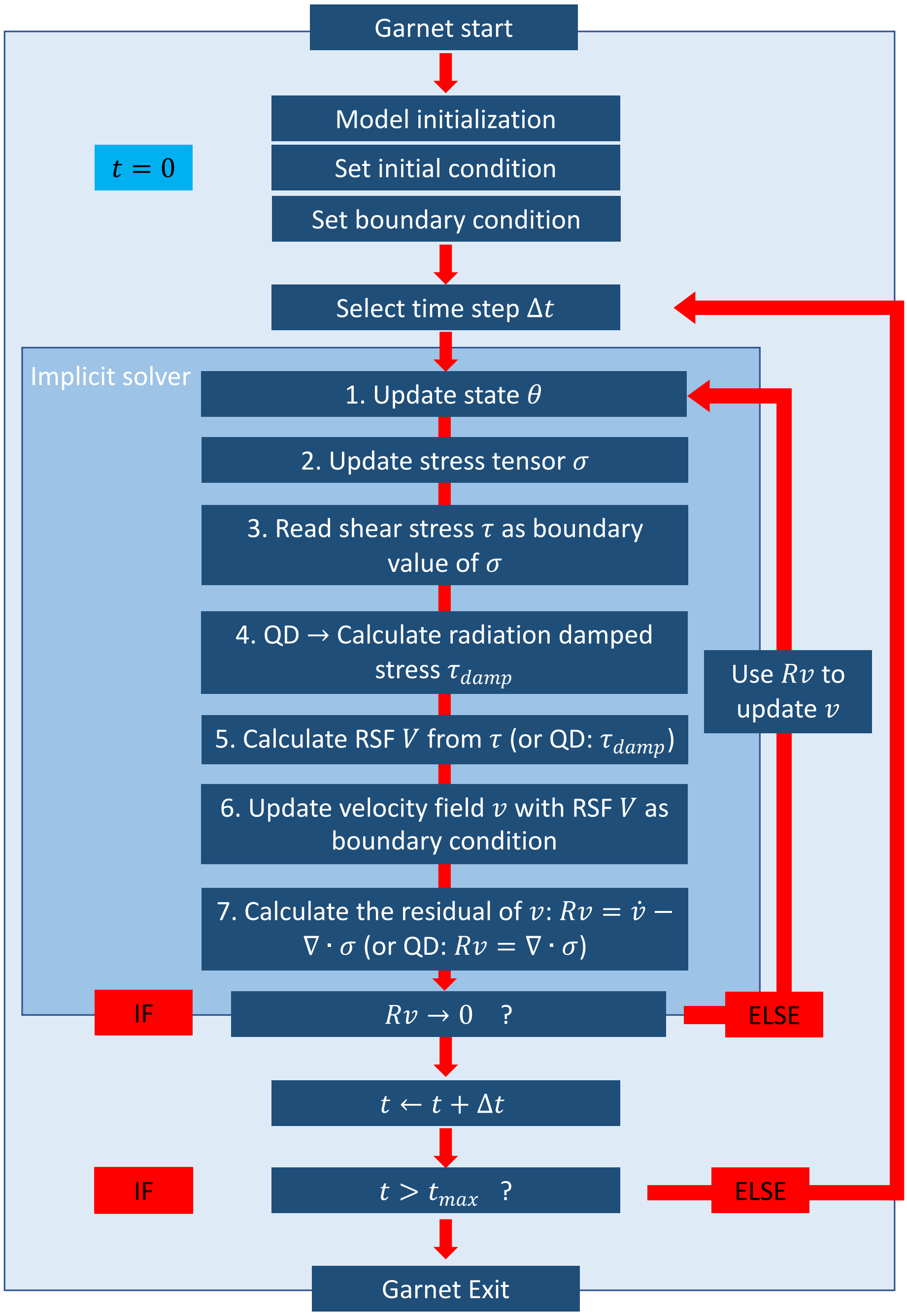

Figure S2. Flowchart of the numerical algorithm: QD models share most steps with FD models in common, steps peculiar for the QD approach are labeled with "QD" closed in the parentheses (steps 4, 5 and 7).

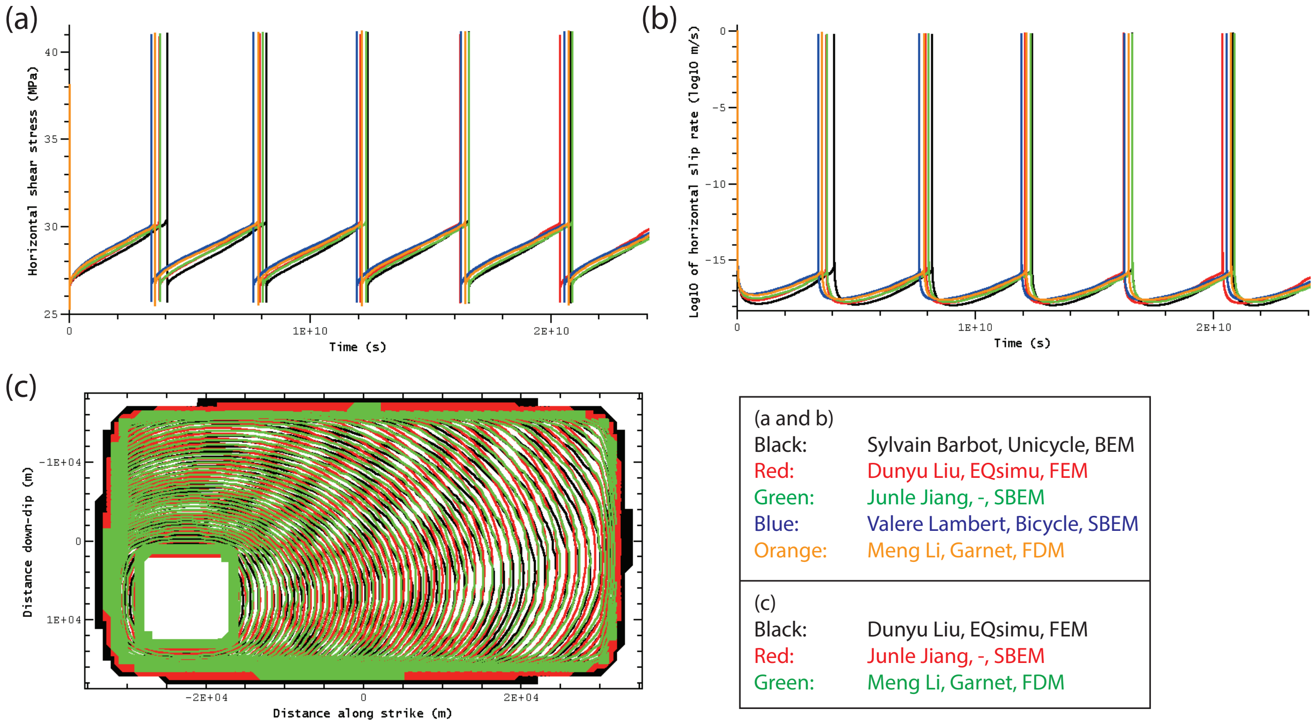

Figure S3. Code validation: Comparison of *Garnet* and other modelers participated in the 3D QD benchmark BP4-QD. (a and b) Long-term time series of slip rate and shear stress (respectively) observed at the center of the VW zone. The result of *Garnet* is in orange. (c) Coseismic rupture front propagation of the first event observed on the fault plane, with the results of *Garnet* in green. The usage of colors and their corresponding models (modeler, model name, method) are summarized in the bottom right box. (Generated by the SEAS online platform, `http://scecdata.usc.edu/cvws/seas/`.)

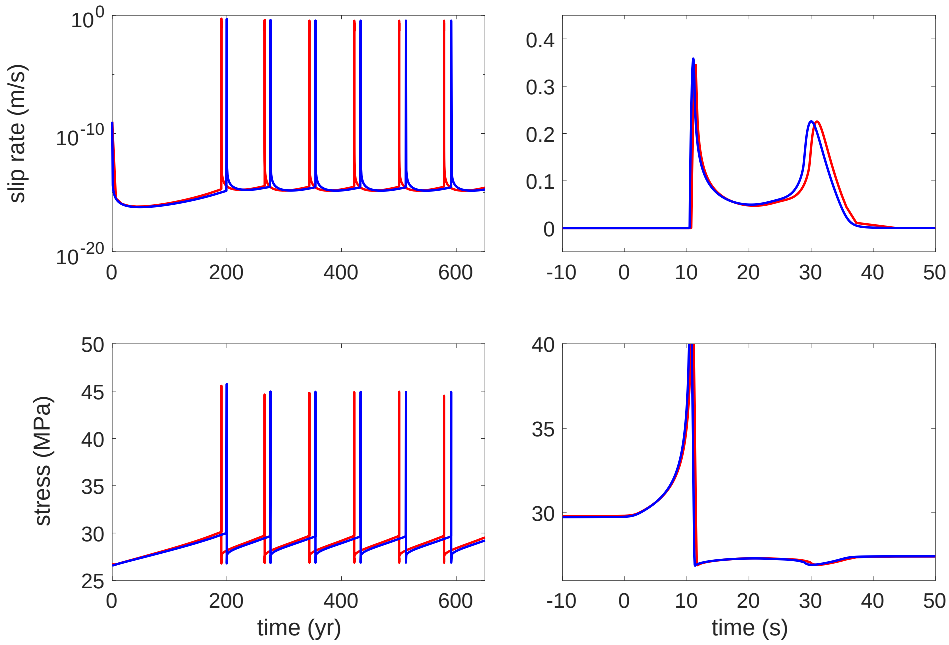

Figure S4. Code validation: comparison between Garnet and BIcyclE results in the 2D QD benchmark BP1-QD. Left: The long term time series of slip rate and shear stress at depth of 7.5 km from BICyclE code (blue) and *Garnet* (red). Right: The coseismic time series of slip rate and shear stress at the same depth. The time origin is reset to the rupture initiation time of the third event for better comparison. (Data available via the SEAS online platform, `http://scecdata.usc.edu/cvws/seas/`.)
